# DNA origami-based shape IDs for single-molecule nanomechanical genotyping

**DOI:** 10.1038/ncomms14738

**Published:** 2017-04-06

**Authors:** Honglu Zhang, Jie Chao, Dun Pan, Huajie Liu, Yu Qiang, Ke Liu, Chengjun Cui, Jianhua Chen, Qing Huang, Jun Hu, Lianhui Wang, Wei Huang, Yongyong Shi, Chunhai Fan

**Affiliations:** 1 Division of Physical Biology and Bioimaging Center, Shanghai Synchrotron Radiation Facility, CAS Key Laboratory of Interfacial Physics and Technology, Shanghai Institute of Applied Physics, Chinese Academy of Sciences, PO Box 800-204, Shanghai 201800, China; 2 Key Laboratory for the Genetics of Developmental and Neuropsychiatric Disorders (Ministry of Education), Bio-X Institutes, Shanghai Jiao Tong University, Shanghai 200030, China; 3 Key Laboratory for Organic Electronics and Information Displays (KLOEID), Institute of Advanced Materials (IAM), School of Materials Science and Engineering, Nanjing University of Posts and Telecommunications, 9 Wenyuan Road, Nanjing 210046, China

## Abstract

Variations on DNA sequences profoundly affect how we develop diseases and respond to pathogens and drugs. Atomic force microscopy (AFM) provides a nanomechanical imaging approach for genetic analysis with nanometre resolution. However, unlike fluorescence imaging that has wavelength-specific fluorophores, the lack of shape-specific labels largely hampers widespread applications of AFM imaging. Here we report the development of a set of differentially shaped, highly hybridizable self-assembled DNA origami nanostructures serving as shape IDs for magnified nanomechanical imaging of single-nucleotide polymorphisms. Using these origami shape IDs, we directly genotype single molecules of human genomic DNA with an ultrahigh resolution of ∼10 nm and the multiplexing ability. Further, we determine three types of disease-associated, long-range haplotypes in samples from the Han Chinese population. Single-molecule analysis allows robust haplotyping even for samples with low labelling efficiency. We expect this generic shape ID-based nanomechanical approach to hold great potential in genetic analysis at the single-molecule level.

## Abstract (web summary)

Atomic force microscopy allows for the imaging of molecules at a nanometre resolution. Here the authors combine AFM with self-assembling DNA origami structures to detect single-nucleotide polymorphisms and determine haplotypes.

State-of-the-art genetic analysis is predominantly based on fluorescence imaging, which nevertheless has an optical resolution limit^1^,^2^,^3^,^4^,^5^,^6^. As an alternative yet powerful approach, atomic force microscopy (AFM)-based nanomechanical imaging is distinct in its high-resolution power in ambient conditions^7^. Direct reading of genetic information using AFM has long been a dream since its invention^8^. Although AFM in principle has much higher resolution than optical microscopy and even superresolution microscopy^9^, its application in genetic analysis remains to be limited. Unlike fluorescence imaging that has a spectrum of wavelength-specific fluorophores or fluorescent nanoparticles, the lack of shape-specific labels for AFM largely restrict site-specific labelling for distinctive visualization^10^,^11^,^12^. Previous studies have well documented that self-assembled DNA origami nanostructures with arbitrary shapes can be reliably fabricated by folding a long viral M13 genomic DNA with ∼200 short complementary staple strands^13^,^14^,^15^,^16^,^17^. By using DNA origami as the soluble substrate, Yan and colleagues^17^ developed nanoscale chips for hybridization detection of nucleic acids. We were thus motivated to repurpose differentially shaped origami nanostructures as unique nanomechanical shape IDs for ‘multi-colour' labelling of genomic DNA. As a proof-of-concept, we explored high-resolution genetic phasing of haplotypes using this nanomechanical imaging method.

The human genome consists of two copies of homologous chromosomes deriving from the farther and the mother, respectively. As a haplotype is the combination of alleles at multiple loci along one chromosome, the context of variations occurring on a haplotype has profound effects on the expression and regulation of genes, and even the aetiology of human diseases^18^,^19^. Especially, revealing long-range haplotype information holds great promise for identifying genetic causal variants of complex disorders^20^,^21^,^22^. However, despite rapid advances in next-generation sequencing (NGS) technologies^23^, along with the international collaborative efforts on constructing a haplotype map of the human genome (for example, the HapMap Project^24^ and the 1000 Genomes Project^25^), our understanding of the diploid nature of the genome in most studies remains limited. Among mainstream haplotyping approaches^6^,^21^,^22^,^26^,^27^, imaging-based three methods are highly attractive, as their direct reading feature complements NGS in rapid assembly of short-reads of several hundred base pairs and provides feasibility and effectiveness in targeted genomic region studies. Several elegant studies have demonstrated the effectiveness of fluorescence imaging in haplotyping^28^,^29^. Nevertheless, the diffraction limit of 200–300 nm restricted the resolution to be of ∼1,000 bp. By taking genetic phasing as a test bed, we herein demonstrated that the shape ID-based nanomechanical imaging provided a powerful method for haplotyping with remarkably improved resolution and at the single-molecule level.

## Results

### Design and fabrication of DNA origami-based shape IDs

**Figure 1:**
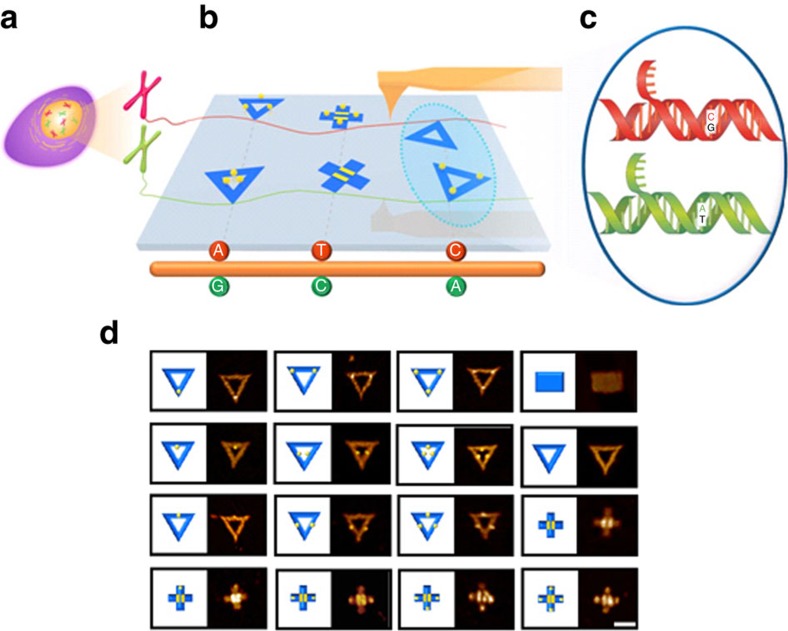
Schematic illustration of AFM-based single-molecule nanomechanical haplotyping with DNA origami shape IDs. (**a**,**b**) Diploid genomic DNA extracted from genetic samples, which was site-specifically labeled with ‘multi-colour' shape IDs. The two alleles of each SNP are labeled with different shape IDs. (**c**) Origami shape IDs serve as ‘magnifying lens' to translate individual SNPs to origami nanostructures of several tens of nanometers. Consequently, the haplotype of this genomic DNA can be directly imaged under AFM. (**d**) Design and AFM images of sixteen shape IDs using DNA origami decorated with or without STV. Scale bar, 100 nm.

**Figure 2:**
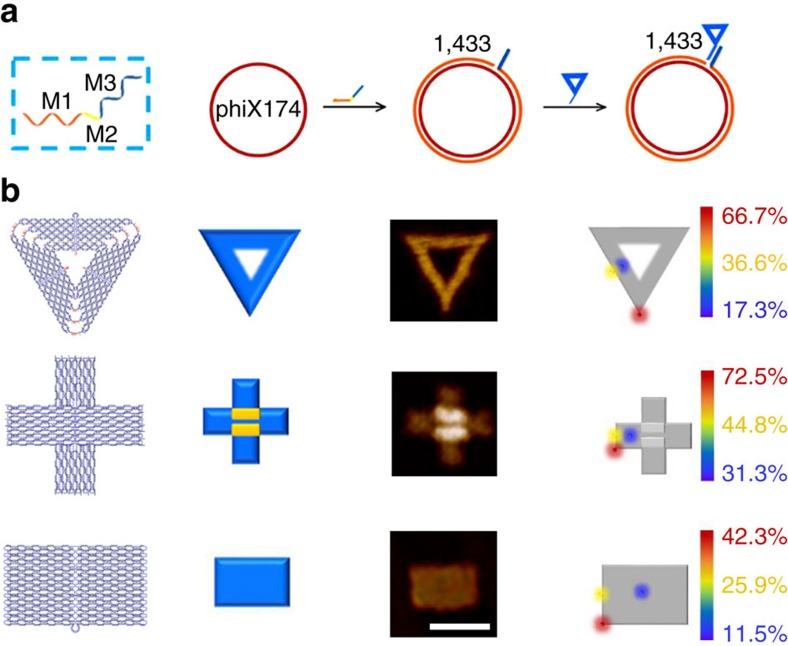
Origami shape IDs for ‘multi-colour' nanomechanical imaging. (**a**) Schematic showing of the three-block ‘mediator' DNA strand (M-strand) in labelling of phiX 174 with shape IDs. Left, M-strand is shown with three function blocks. M1 block (orange-coloured region) for site-specific hybridization and extension with the template, M2 spacer block (yellow-coloured region) and a M3 block (blue-coloured region) for capturing shape IDs. Right, the phiX 174 template is first annealed with the M-strand on the site 1,433, then M1 block serves as allele-specific primer to initiating DNA extension in the presence of polymerase, which turns the ssDNA template into dsDNA that is more visible under AFM imaging. M3 block is complementary to a short strand M3′ on each corresponding shape ID. (**b**) Schematic and AFM images with the labeling efficiency of individual shape IDs. Left to right: design (column 1), schematic (column 2) and AFM images (column 3) of triangular-, cross- and rectangular- shaped DNA origami IDs. Scale bar, 100 nm. Position effects on the hybridization efficiency of shape IDs on the site 1,433 of phiX174 (column 4, see also in Supplementary Figs 2–10).The efficiency is the highest at the point position (‘hotspot', marked in red). The edge middle and inner sites are marked in yellow and blue, respectively.

We fabricated a set of shape IDs by using DNA origami designs (Figs 1 and 2, and Supplementary Fig. 1). The basic elements are triangular, cross and rectangular shapes, which are readily distinguishable under AFM imaging. To establish that this shape ID system can specifically target gene sequences, we employed a single-stranded (ss-) bacteriophage phiX 174 DNA with a covalently closed circularity genome of 5,386 nucleotides as the testbed for genetic analysis. The phiX 174 template was first annealed with a three-block ‘mediator' DNA strand (M-strand) that has an M1 block for complementary hybridization with the template, an M2 spacer block and an M3 block for capturing shape IDs (Fig. 2a). Upon hybridization with the template, M1 serves as the primer to initiating DNA extension in the presence of polymerase, which turns the ssDNA template into double-stranded (ds) DNA that is more visible under AFM imaging^8^. The M3 block is complementary to a short strand M3′ that is carried on each corresponding shape ID.

### Genotyping using shape IDs

To test the hybridizability of shape IDs, which is critically important for their targeting efficiency, we interrogated both the shape effect of origami and the position effect of M3′ on the labelling efficiency of shape IDs on the site 1,433 of phiX 174 (that is, hybridization efficiency between M3′ and M3). As a general trend, the labelling efficiency using the triangular and the cross origami is much higher than that using the rectangular one. The labelling efficiency is also strongly dependent on the M3′ position, which decreases in the order of corner, edge middle and inner space (Fig. 2b). Hence, both the shape of the origami and the position of M3′ on the origami greatly affect the hybridizability of shape IDs. Remarkably, we found corner positions were ‘hotspots' for on-origami hybridization (with the efficiency of 72.5% and 66.7% for cross and triangular, respectively), which coincides with the fact that these positions have minimal steric effects, and are least perturbed by the electrostatic force field imposed by hundreds of negatively charged DNA strands in an origami (Supplementary Figs 2–10). Further studies revealed that placing multiple M3′ strands on corners of a single origami (three for triangular and four for cross) led to even greater labelling efficiency up to 85%, an effect arising from increased M3–M3′ inter-strand collision probability (Supplementary Figs 11 and 12, and Supplementary Table 1). Hence, we employed highly hybridizable triangular and cross shape IDs with multiple M3′ for subsequent genetic analysis.

**Figure 3:**
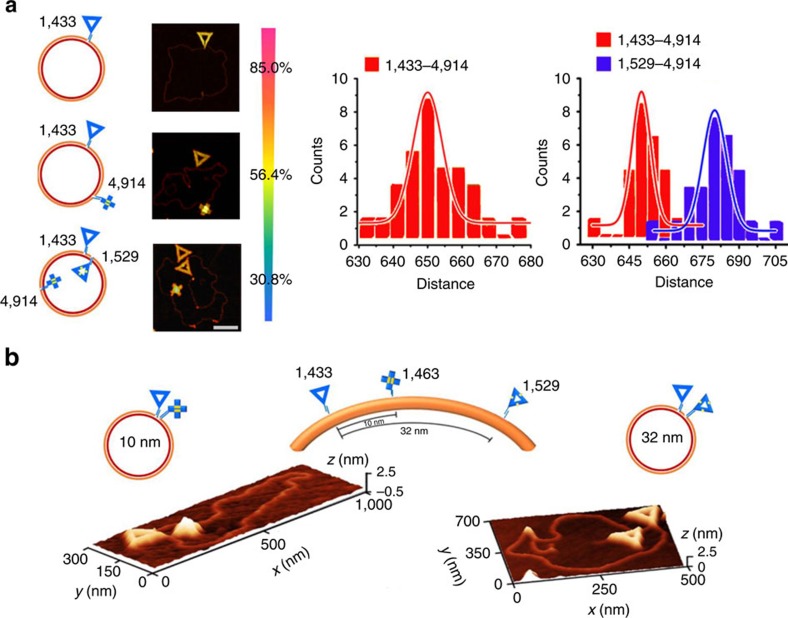
‘Multi-colour' nanomechanical imaging and genotyping resolution of origami shape IDs. (**a**) ‘Multi-colour' imaging of phiX174 with multiple shape IDs and their distance analysis. Left, three different shape IDs, triangular (corresponding to site 1,433), triangular with STVs (corresponding to site 1,529) and cross (corresponding to site 4,914), are site-specifically labelled on phiX 174 with different labelling efficiency (85% for the single-site labelling, 56.4% for the two-site labelling and 30.8% for the three-site labelling). Scale bar, 200 nm. Right, histograms for the counts of shape IDs as a function of distance between site 1,433, 1,529 and 4,914 in phiX. As for two-site labelling (left), the measured distance between site 1,433 and 4,914 is 650 nm, which is in good agreement with calculated distance 648 nm. As for three-site labelling (right), the measured distances between site 1,433, 1,529 and 4,914 are 652 nm (blue) and 682 nm (red), which are in good agreement with the calculated distances 648 and 680 nm. Also see Supplementary Figs 14 and 15. (**b**) Schematic showing and three-dimensional AFM topographic images of triangular- (corresponding to site 1,433), cross- (corresponding to site 1,463) and STV-decorated triangular- (corresponding to site 1,529) shaped IDs for labelling phiX 174. Left, the contour length between two labelled sites (1,433 and 1,463) is ∼10 nm (30 bp). Right, the contour length between two labelled sites (1,433 and 1,529) is ∼32 nm (96 bp).

Having substantiated the specific gene-targeting ability of individual shape IDs, we next explored differential labelling of a single template using multiple shape IDs. We first expanded the three-element set of shapes by using streptavidin (STV)-decorated origami nanostructures (Supplementary Fig. 13a). By exploiting the addressability of DNA origami, we site-specifically anchored different numbers of STV on prescribed positions carrying biotin tags. STV of ∼4 nm is readily visible as small dots on the origami, which remarkably distinguishes STV-decorated shape IDs from non-decorated ones. Three different shape IDs, triangular, triangular with STV and cross were then chosen to target the sites of 1,433, 1,529 and 4,914 on phiX 174, respectively. We employed multiple ‘mediator' DNA strands to introduce the specific M3 capturing sequences. We found that multiple M1 sequences could initiate simultaneous extension from different sites of phiX 174 in the presence of polymerase, which turned the template into a dsDNA circle similar to the single M1 initiation, albeit with a lower efficiency (Supplementary Figs 13b and 16–20 and Discussion). Importantly, multiple shape IDs can be site-specifically labelled on phiX 174, as illustrated in AFM images (Fig. 3a and Supplementary Figs 18 and 19). We also note that contour lengths between adjacent sites coincide well with those predicated from their base numbers (0.34 nm for one base) for labels at either two or three sites (Supplementary Figs 14 and 15). Of note, whereas the labelling efficiency decreases from single site (∼85%) to two sites (∼56.4%) and three sites (∼30.8%), the single-molecule nature of this nanomechanical analysis allows reliable genotyping even at a low efficiency of 30.8% (Fig. 3a and Supplementary Fig. 20). Analogous to ‘multi-colour' fluorescence imaging-based genetic analysis, differential labelling of multiple shape IDs should greatly improve the genotyping ability in both increasing the accuracy and reducing the labour and time cost. Moreover, accurate distance information between single-nucleotide polymorphism (SNP) sites can be readily obtained from single-molecule-based statistical analysis (Fig. 3a).

We next explored the genotyping resolution of this shape ID-based single-molecule analysis. To approach the sub-100 bp resolution limit, we chose three sites (1,433, 1,463 and 1,529) on phiX 174 for labelling of shape IDs, which are separated by 30 bp (∼10 nm) and 96 bp (∼30 nm), respectively. Although DNA origami is ∼100 nm^2^ in size, it is easy to resolve its centre and realize nanometre resolution given its rigid structure. Indeed, AFM imaging revealed that these sites could be readily resolved by labelled shape IDs (Fig. 3b). We note that this 10 nm resolution is the highest in imaging-based haplotyping.

**Figure 4:**
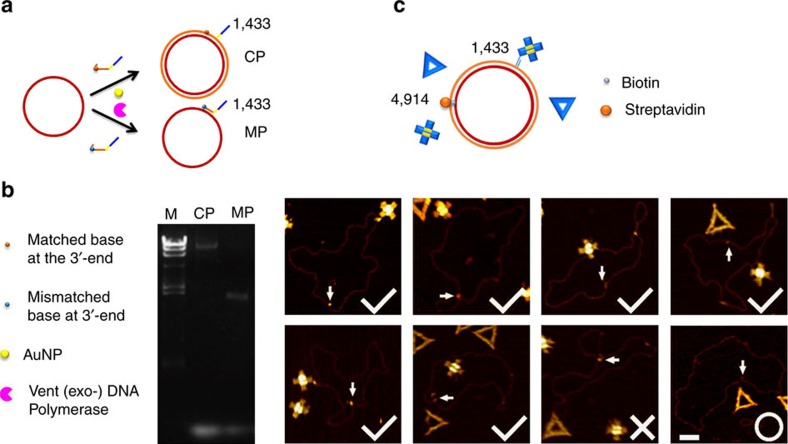
Specificity of origami shape IDs. (**a**) Schematic showing of AuNP-assisted allele-specific primer extension of phiX 174 by a fully complementary primer (CP) or no extension by a mismatched primer (MP), the single mismatched base of which is at the 3′-end. (**b**) Agarose gel electrophoresis validated that CP could initiate the extension of template ssDNA into dsDNA, whereas MP could not. M, DL 15, 000 marker. (**c**) Upper: schematic showing of shape ID-based haplotyping. The site 1,433 is labelled with two M-strands with or without a single-base mismatch, which correspond to a triangular- and a cross-shaped ID, respectively. The site 4,914 is hybridized with a biotin-modified primer, which serves as the reference point. Lower: AFM images show that phiX 174 is correctly labelled with cross-shaped IDs (marked by tick), wrongly labelled with triangular (marked by cross), or with no labelling (marked by circle). The site 4,914 labelled with STV is marked with an arrowhead. The yield of specific labelling of shape IDs is ∼88%. Scale bar, 100 nm.

We further performed genotyping of SNPs, which forms the basis for many types of genetic analysis. Our previous studies showed that gold nanoparticles (AuNPs) could effectively inhibit mismatched binding^30^,^31^. Hence, we employed AuNPs to assist discrimination of single-base mismatches in primer extension. To mimic polymorphic sites, we designed two primers with or without a single-base mismatch site at the 3′-end (Fig. 4a). Gel electrophoresis revealed that only the fully complementary primer could initiate primer extension to form the dsDNA circle, whereas the extension of single-base mismatched one was completely inhibited in the presence of AuNPs (Fig. 4b and Supplementary Fig. 23). To substantiate the specificity in AFM imaging, we chose the site 1,433 on phiX 174 to test two primers with or without a single-base mismatch, which correspond to a triangular and a cross shape ID, respectively (Fig. 4c). The site 4,914 was hybridized with a biotin-modified primer that could bind to STV, which served as the reference point. Statistical analysis of AFM images revealed that 88.0% of the phiX 174 template was correctly lableled with the cross shape ID, which confirms the high stringency of AuNPs-enhanced primer extension for SNP genotyping using shape IDs (Supplementary Fig. 25)^31^.

### Single-molecule haplotyping in real samples

**Figure 5:**
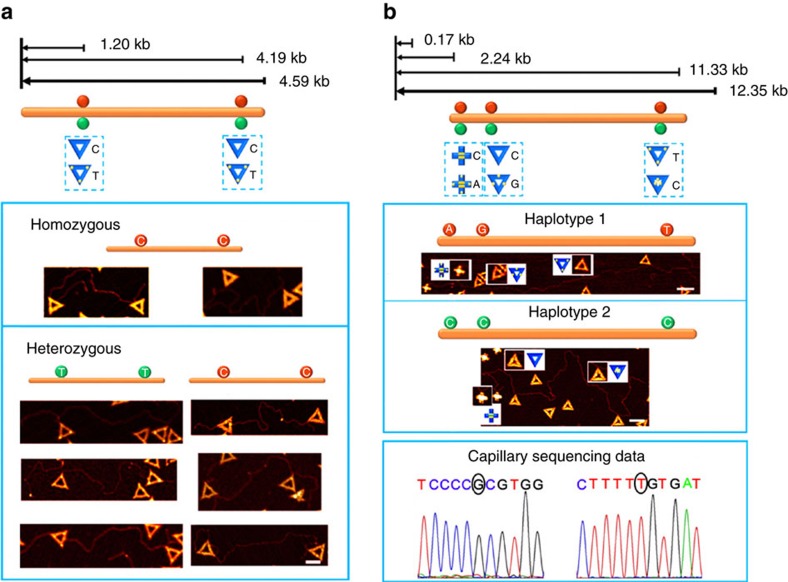
Single-molecule haplotyping. (**a**) Upper, schematic showing of genotypes and relative locations of two SNPs on a 4.6 kb region. SNP loci rs17038640 (C/T) and rs4676487 (C/T) are 1.20 and 4.19 kb away from one end of the region. Lower, AFM images show that the genomic DNA of one sample is homozygous at both loci (C–C) and the genomic DNA of the other three samples is heterozygous at both loci (T–T and C–C). For each SNP locus, the triangular- and the STV-decorated triangular-shaped IDs are employed to label the alleles C and T, respectively. Scale bar, 100 nm. (**b**) Single-blinded test for haplotying *P53* gene. Upper, schematic showing of a 12 kb region of the human *p53* gene located on the chromosome 17. Each origami shape ID corresponds to a specific allele. For example, for the SNP 1, A and C allele correspond to cross origami with or without STVs, respectively. Middle, AFM images for the haplotypes of these three SNPs. Haplotype 1 contains A–G–T and haplotype 2 contains C–C–C. Lower, when the first SNP was A on one sequence, the other SNPs obtained from independent capillary sequencing were G and T, which was consistent with that from the nanomechanical imaging in haplotype 1. Scale bar, 200 nm.

The high-resolution, high-specificity and ‘multi-colour' imaging ability of shape IDs opens new opportunities for single-molecule genotyping and haplotyping. As a first example, we phased a 4.6 kb region on the chromosome 3 from the Han Chinese population. There are two SNP loci in this region, that is, rs17038640 (C/T) and rs4676487 (C/T). For each locus, we employed triangle with or without STV to visualize SNPs under AFM. Genomic DNA extracted from human blood samples was subjected to amplification using exonuclease-assisted and AuNP-enhanced long-range PCR (Supplementary Figs 26 and 27), generating linear dsDNA for AFM imaging. The two loci locate 1.2 and 4.19 kb from the 5′-end of the 4.6 kb fragment, which were labelled with triangular shape ID with or without streptavidin. AFM images revealed that the genomic DNA of one sample was homozygous at both loci and the genomic DNA of the other three samples was heterozygous at both loci. These direct read sequences of each allele (Fig. 5a) were confirmed using capillary sequencing^31^.

As a further illustration of the ability to phase haplotypes in the long range, we genotyped a 34 kb region associated with age-related macular degeneration (AMD), which is the leading cause of blindness worldwide. This region carries seven SNP alleles on the chromosome 10 from the Han Chinese population. To avoid the inefficiency in amplifying this long region, we split it into four overlapping fragments, each having two or three SNP loci (Supplementary Fig. 28). Each fragment has at least one SNP locus (that is, ‘joint locus') that is heterozygously shared by its adjacent fragment, which can be used for subsequent fragment connection. As an example, SNP-2 (rs11200630) was used as the joint locus for fragment 1 and 2 to connect these two fragments. By using shape ID-based AFM imaging, we could genotype SNP loci on each fragment via direct reading. Connection of the four fragments reconstructs a continuous haplotype for this 34 kb region. We found that all the seven SNPs are heterozygous in the sample and two haplotypes were directly read using AFM (Supplementary Fig. 29), which were also supported by the capillary sequencing results^31^.

Having established the ability of shape ID-based nanomechanical imaging for reliable phasing known haplotypes of AMD, we further explored to haplotype a 12 kb region of the human *p53* gene located on the chromosome 17 using a single-blinded test (Fig. 5b and Supplementary Fig. 30). The haplotype of this region has not been shown in the literature. We first sequenced this region in a sample of Han Chinese origin by using capillary sequencing and found three heterozygous SNP sites: C/A at rs12951053, C/G at rs1042522 and C/T at rs17882227. By using six different shape IDs to label these sites, we obtained the haplotype of this region (Fig. 5b). We independently haplotyped the same region using our previously reported strategy that combines AuNP-assisted allele-specific PCR with capillary sequencing. Significantly, this single-blinded test showed consistent haplotyping information, which clearly illustrated the reliability of the new imaging-based haplotyping method. Also of note is that the first two SNPs are spaced by 170 bp (∼55 nm), which cannot be resolved using a fluorescence imaging-based haplotyping method due to the limit of optical diffraction^28^,^29^, which highlights the significance of developing high-resolution haplotyping methods.

## Discussion

High-resolution imaging is an ever-increasing desire for genetic analysis given the complexity of the genome, especially for resolving genetic variations in close proximity. AFM-based nanomechanical imaging is intrinsically a high-resolution method that has long been pursued as a promising tool for single-molecule genetic analysis and manipulation^10^,^11^,^12^,^32^,^33^. However, direct visualization of single DNA nucleotides in air is currently not feasible under AFM. Prior efforts to using label-based AFM imaging led to resolution limit of several tens of nanometres due to the lack of appropriate labels with high contrast^12^. In this work, we developed a new strategy using origami shape IDs as ‘magnifying lens' to translate angstrom-sized individual nucleotides to origami nanostructures of several tens of nanometres. We demonstrated that this nanomechanical imaging method reached an ultrahigh haplotyping resolution of 10 nm (30 bp) that far exceeds fluorescence- and even superresolution-based imaging^34^,^35^,^36^. As this new method does not have a theoretical limit, it is possible to design smaller shape IDs (<10 nm) to further increase the resolution, which holds great promise SNP genotyping approaching single-base resolution. We also envision that this technology might provide a direct reading-based route to *de novo* assembly of genomes from short reads obtained from NGS DNA sequencing^23^.

DNA origami is powerful technology that can fold a long ssDNA template to virtually any prescribed shapes or patterns, which have found numerous applications in various areas. Nevertheless, the presence of intense negative electrostatic force field and significant steric effects pose intense limits for on-origami DNA hybridization. Our finding of highly hybridizable ‘hotspots' on points of triangular and cross origami allows us to repurpose DNA origami nanostructures as nanomechanical shape IDs (with a single labelling efficiency of ∼85%). Notably, by combining the shape designing and protein patterning abilities of DNA origami, it is possible to design virtually unlimited numbers of shape IDs for distinguishable AFM imaging (up to 16 as illustrated in this work). This compares favourably with fluorescence imaging (typically four colours) or super-resolution imaging (one to two colours) that are restricted by spectral overlap of fluorophores^36^. In addition, these shape recognition-based nanomechanical imaging has high imaging contrast and is less prone to artefacts due to the absence of bleaching or blinking of fluorophores in fluorescence imaging.

Single-molecule analysis using nanomechanical imaging is insusceptible to artefacts that are often encountered in ensemble assays. We can image individual long genomic DNA with labelled shape IDs and determine the relative locations of the latter from the visualizable contour length of the former. Non-labelled shape IDs remaining on the surface are clearly distinguishable from those labelled on the genomic DNA. More importantly, statistical analysis of multiple genomic DNA strands provides a robust means for genetic analysis, even at a relatively low labelling efficiency in multiplexing. As a demonstration of this single-molecule nanomechanical imaging method, we have phased the haplotypes of a target region associated with AMD in the Han Chinese people and a previously unknown haplotype of a fragment DNA in P53, which were all confirmed by capillary sequencing. We also note that, although the cost, speed and throughput of this single-molecule haplotyping remain to be improved, advances in high-speed and parallel AFM should provide a solution.

The real-world applications of this technology remain constrained by the cost and availability of AFM. The lab-based AFM is expensive and not easy to use or access. However, AFM is in principle a nanomechanical cantilever, which can be mass-produced with low cost and in a highly parallel manner. The development and mass-production of automated AFM should be a key step for the practical utility of single-molecule haplotyping.

In summary, we have repurposed DNA origami as highly hybridizable nanomechanical shape IDs for targeted genetic phasing of long-range haplotypes. The availability of a wide spectrum of distinguishable shape IDs leads to efficient ‘multi-colour' labelling of genomic DNA for robust haplotyping at the single-molecule level. Owing to the magnifying effect of these shape IDs, we can reliably phase haplotypes with an ultrahigh resolution of 10 nm. These DNA origami-based shape IDs also hold great promise as biomolecular barcodes for *in-situ* probing of genetic variations on the genome. With the new advances of AFM in both resolution and throughput, it is also possible to implement direct sequencing of the genome and rapid genetic diagnosis for clinical translation.

## Methods

### Oligonucleotides

Sequences of the oligonucleotides and primers used in this work are available in the Supplementary Information.

### Fabrication of the DNA origami shape IDs

Triangular and cross-shaped DNA origami shape IDs were prepared by annealing the single scaffold stranded M13mp18 with staple strands and capturing strands in 1xTAE-Mg^2+^ (40 mM Tris, 20mM acetic acid, 2mM EDTA and 12.5mM magnesium acetate, pH 8.0) with a ratio of 1:10 from 95°C to room temperature in a rate of 1°C min^−1^ (refs 14, 37). In the experiment where STV is decorated in origami, 10 × excess of STV was added before incubation. All DNA Origami IDss were purified by agarose gel electrophoresis and subsequently extracted using Freeze ‘N Squeeze DNA Gel Extraction Spin Columns.

### PCR reaction

A master mix contains 1.25 ng of template DNA, 0.4 μM each of the forward and reverse PCR primers, 0.25 μl LA Taq (5 units μl^−1^), 2.5 μl of 10 × LA PCR Buffer II (Mg^2+^ plus), 4 μl of dNTP mixture (2.5 mM) in 25 μl reaction volume, which was prepared on ice. One of the PCR primes was modified with 5′-Cy3 label and the other was modified with 5′-phosphorylation. The mixture was annealed in a PCR thermocycler and was subjected to a 2 min hot start at 95 °C, followed by 30 cycles of amplification (98 °C for 10 s, then 68 °C for 15 min). The protocol ends with a final extension step at 72 °C for 10 min.

### Generation of long single-stranded DNA template

To generate the single-stranded DNA, the PCR product was digested. Two microlitres of lambda exonuclease, 3 μl of 10 × lambda exonuclease reaction buffer and purified dsDNA were mixed to 30 μl and incubated in a thermocycler for 6 h at 37 °C. The exonuclease was heat inactivated at 85 °C for 8 min after the digestion.

### AuNPs-enhanced primer extension

The sequences of mediator DNA primers were designed by Primer Premier 5.0 and NUPACK ( www.nupack.org/)^38^. ssDNA was annealed with primers in extension solution (20 μl), which consists of 1 × ThermoPol Reaction Buffer, 0.25 mM each dNTP, 0.75 μM each primer, 4 nM of AuNPs and deionized water. The allele-specific labelling reaction was performed in a PCR thermal machine. The extension was initiated from 95 °C (2 min) and annealed to 25 °C at room temperature. Vent (exo-) DNA Polymerase was added and stay at 60 °C for 45 min. The linear amplification products were purified from agarose gel to remove excess primers, nucleotides, enzymes, salts and other impurities, and ready for hybridization.

### Hybridization of target DNA with STV or origami shape IDs

Biotinylated or mediator primers labelled target DNA was incubated with excessive amounts of STVs or origami shape IDs at 37 °C for 30 min.

### AFM imaging

Three-microlitre solution of the samples were deposited on a freshly cleaved mica surface and left to adsorb to the surface for 3 min, then the mica surface was slowly rinsed with water three times (each time with 100 μl water) to wash away the salt. Finally, the mica surface was dried with a mild air stream by an ear-washing bulb and was imaged with a MultiMode 8 AFM with NanoScope V Controller (Bruker, Inc.) in air under tapping mode.

### Single-molecule image analysis

The contour length of DNA molecules and the distances between every two sites labelled by origami IDs or STVs were measured by ImageJ (National Institutes of Health, Bethesda, Maryland, USA, http://imagej.nih.gov/ij/).

### Data availability

The data supporting the main findings of this study are available within the main article and its Supplementary Information (Supplementary Figs 1–30 and Supplementary Tables 1–7) or from the authors upon request.

## Additional information

**How to cite this article:** Zhang, H. *et al*. DNA origami-based shape IDs for single-molecule nanomechanical genotyping. *Nat. Commun.* **8,** 14738 doi: 10.1038/ncomms14738 (2017).

**Publisher's note**: Springer Nature remains neutral with regard to jurisdictional claims in published maps and institutional affiliations.

## Supplementary Material

Supplementary Information Supplementary Figures and Supplementary Tables
