# Supplementary material for: DNA origami-based shape IDs for single-molecule nanomechanical genotyping
Source: Nat Commun. 2017 Apr 6;8:14738. doi: 10.1038/ncomms14738 (PMC5384221; doi:10.1038/ncomms14738)
Supplement: Supplementary Information — Supplementary Figures and Supplementary Tables [file ncomms14738-s1.pdf]

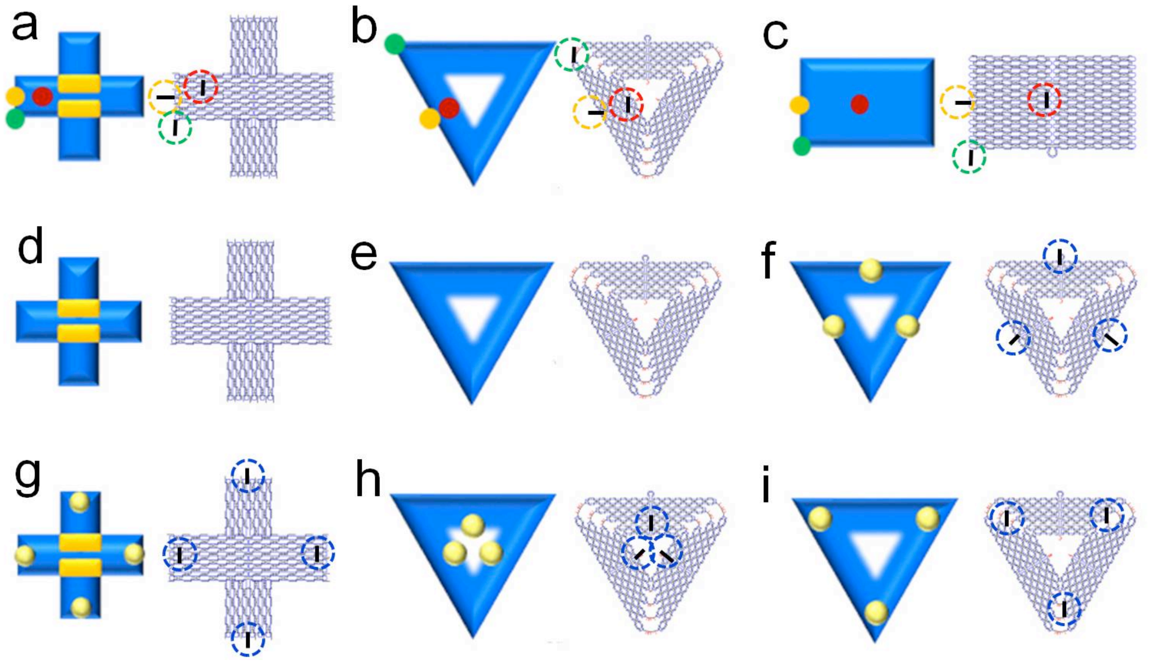

Supplementary Figure 1 |Schematic illustration of origami shape IDs' design. (a) The cross shaped DNA origami IDs with one M3' single strand at different positions; **(b)** the triangular shaped DNA origami IDs with one M3' single strand at different positions; **(c)** the rectangular shaped DNA origami IDs with one M3' single strand at different positions. **(d-i)** Six shape IDs were fabricated by cross, triangular shaped DNA origami and STV modified different shaped DNA origami at different positions.

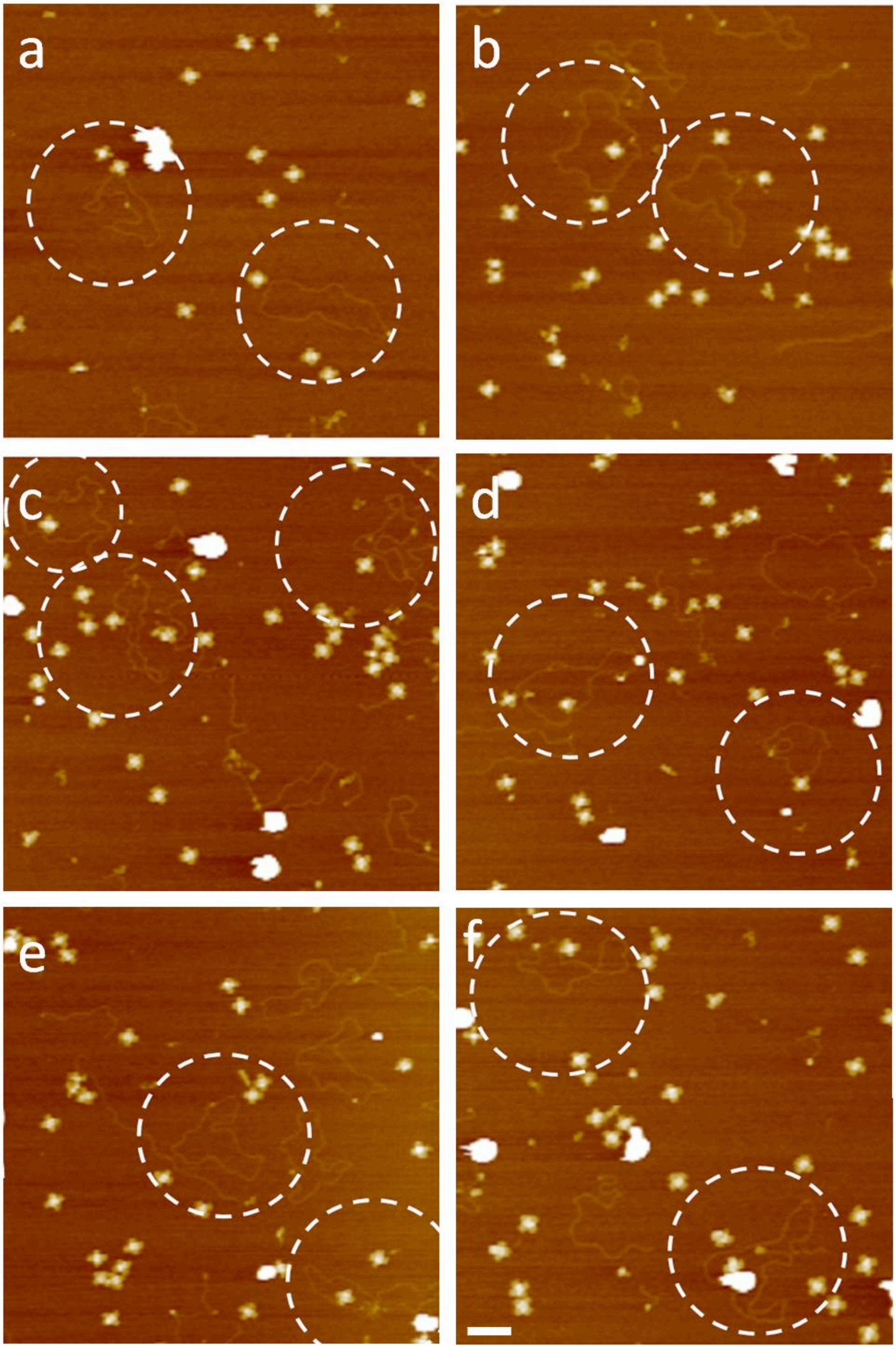

Supplementary Figure 2 | (a-f) The site-specific single labeling of phiX 174 by cross shaped ID with one M3' strand at the corner point of origami. Scale bar: 200 nm.

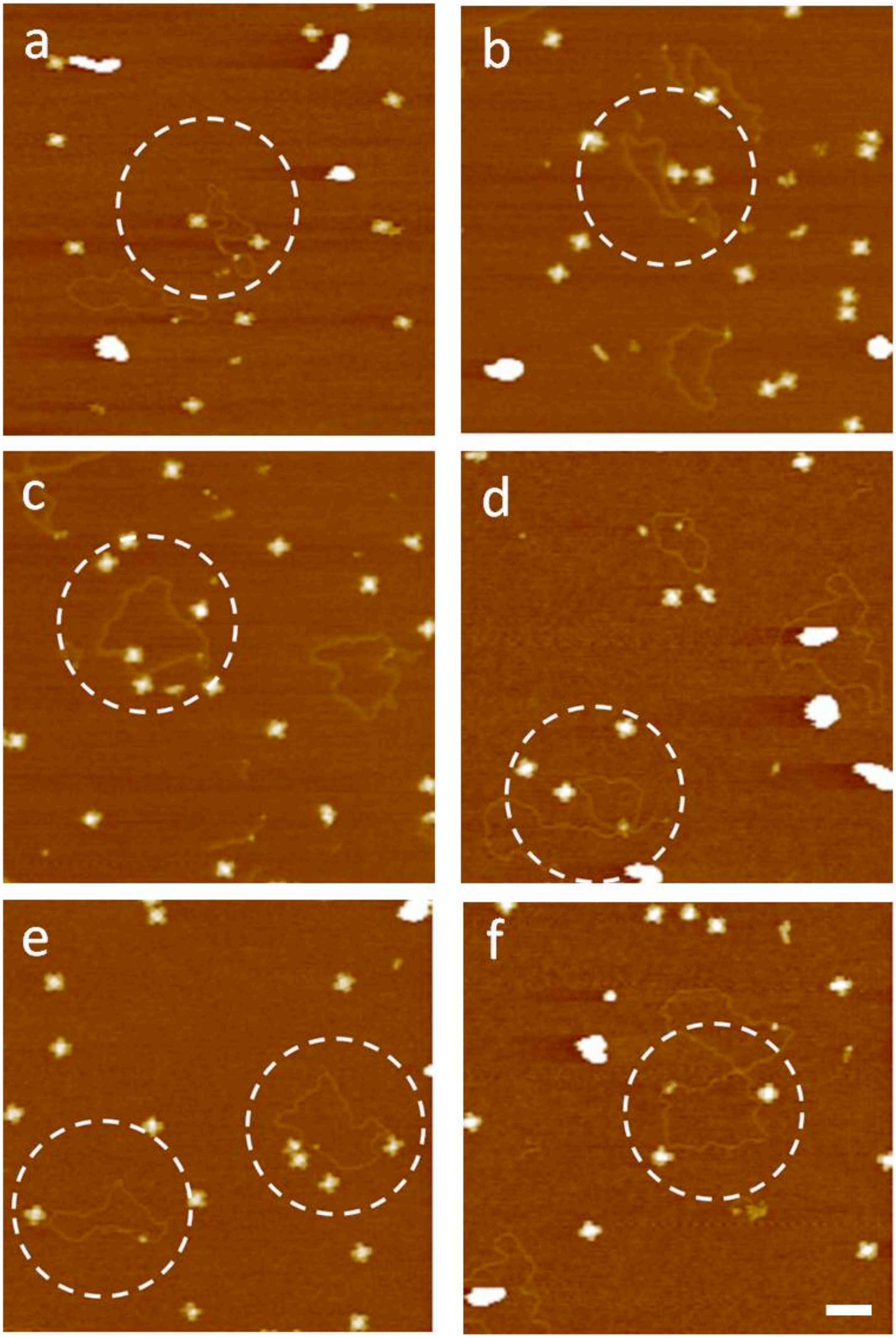

Supplementary Figure 3 | (a-f) The site-specific single labeling of phiX 174 by cross shaped ID with one M3'strand at the edge middle of origami. Scale bar: 200 nm.

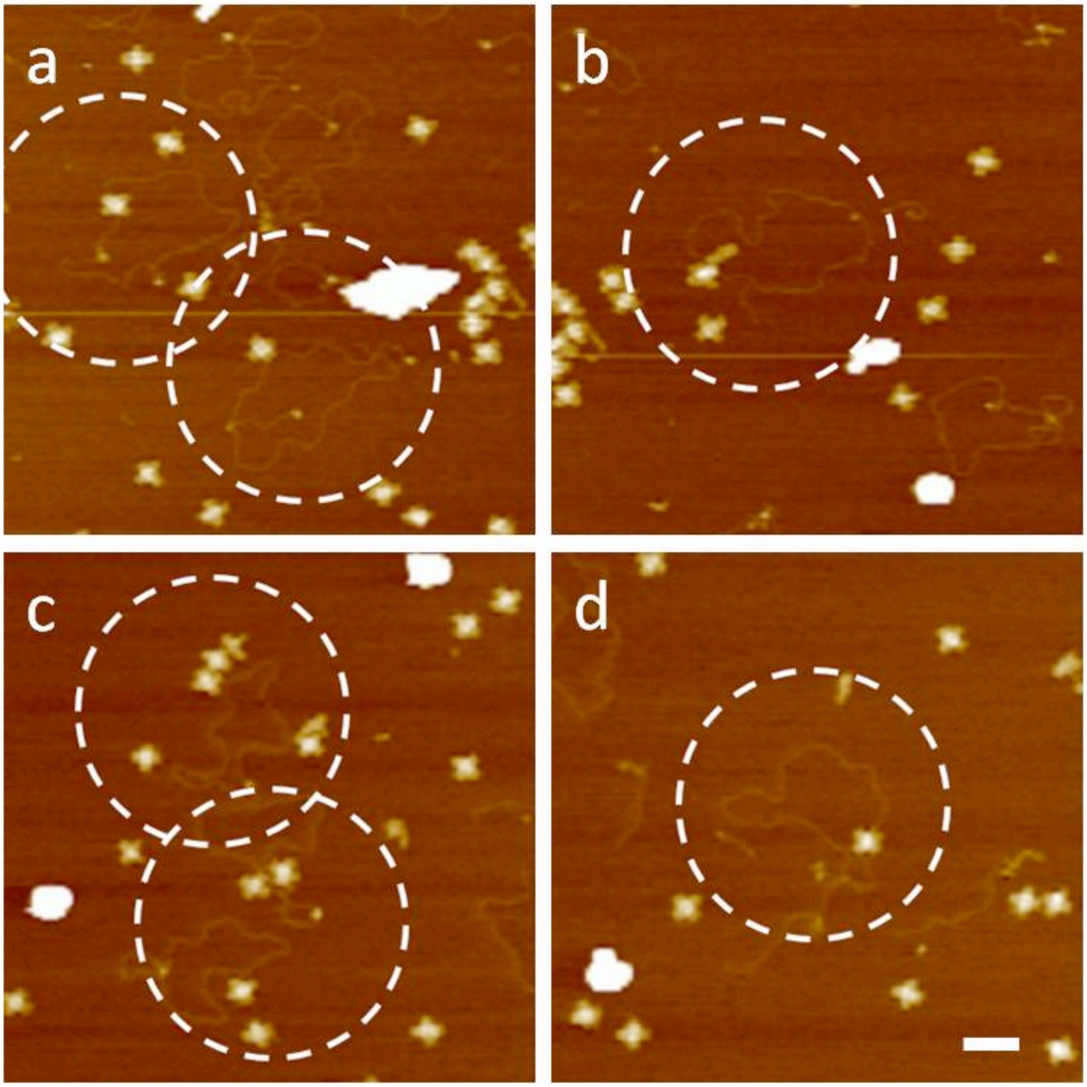

Supplementary Figure 4 | (a-d) The site-specific single labeling of phiX 174 by cross shaped ID with one M3' strand at the inner of origami. Scale bar: 200 nm.

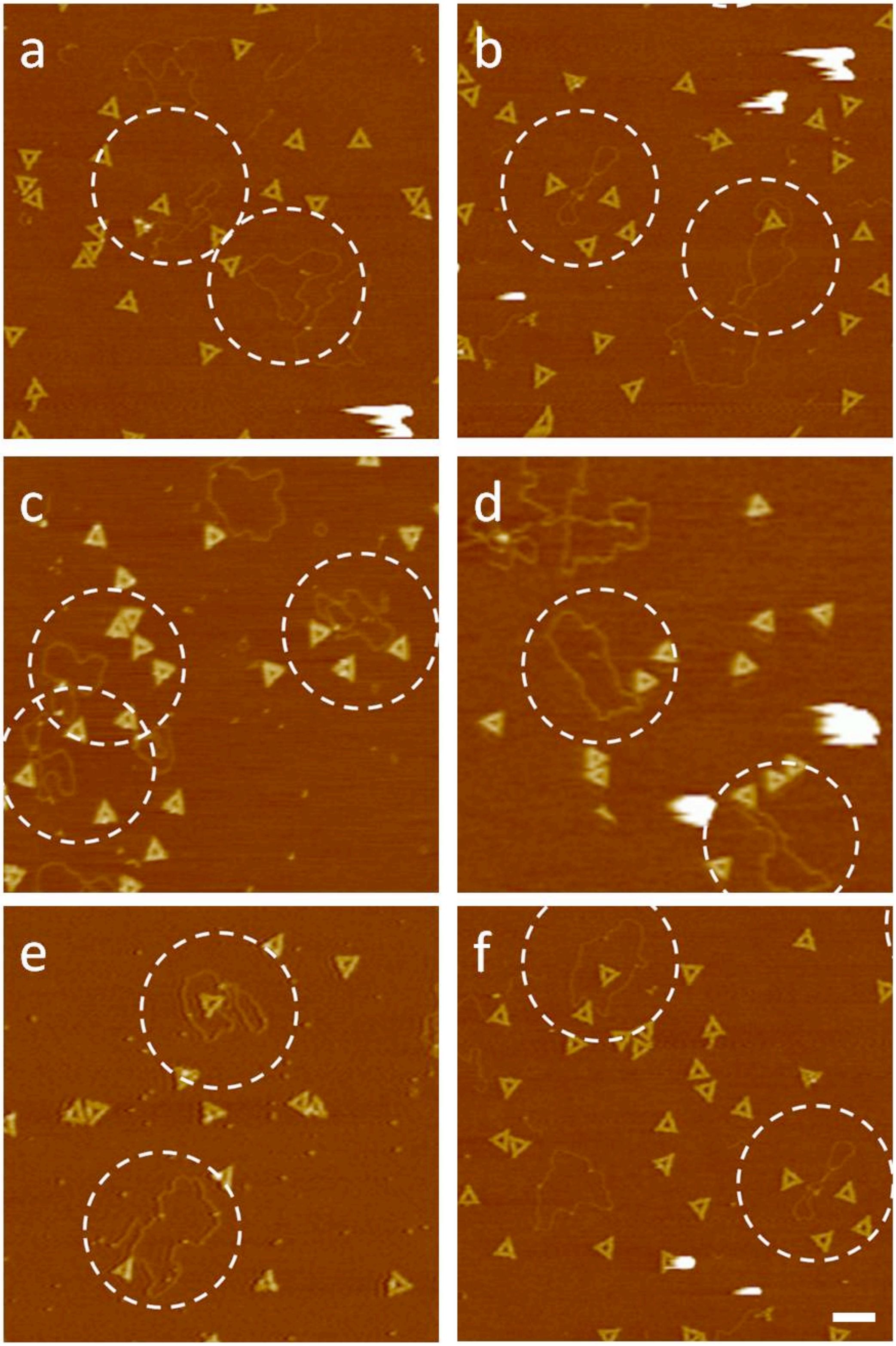

Supplementary Figure 5 | (a-f) The site-specific single labeling of phiX 174 by triangular shaped ID with one M3' strand at the corner point of origami. Scale bar: 200 nm.

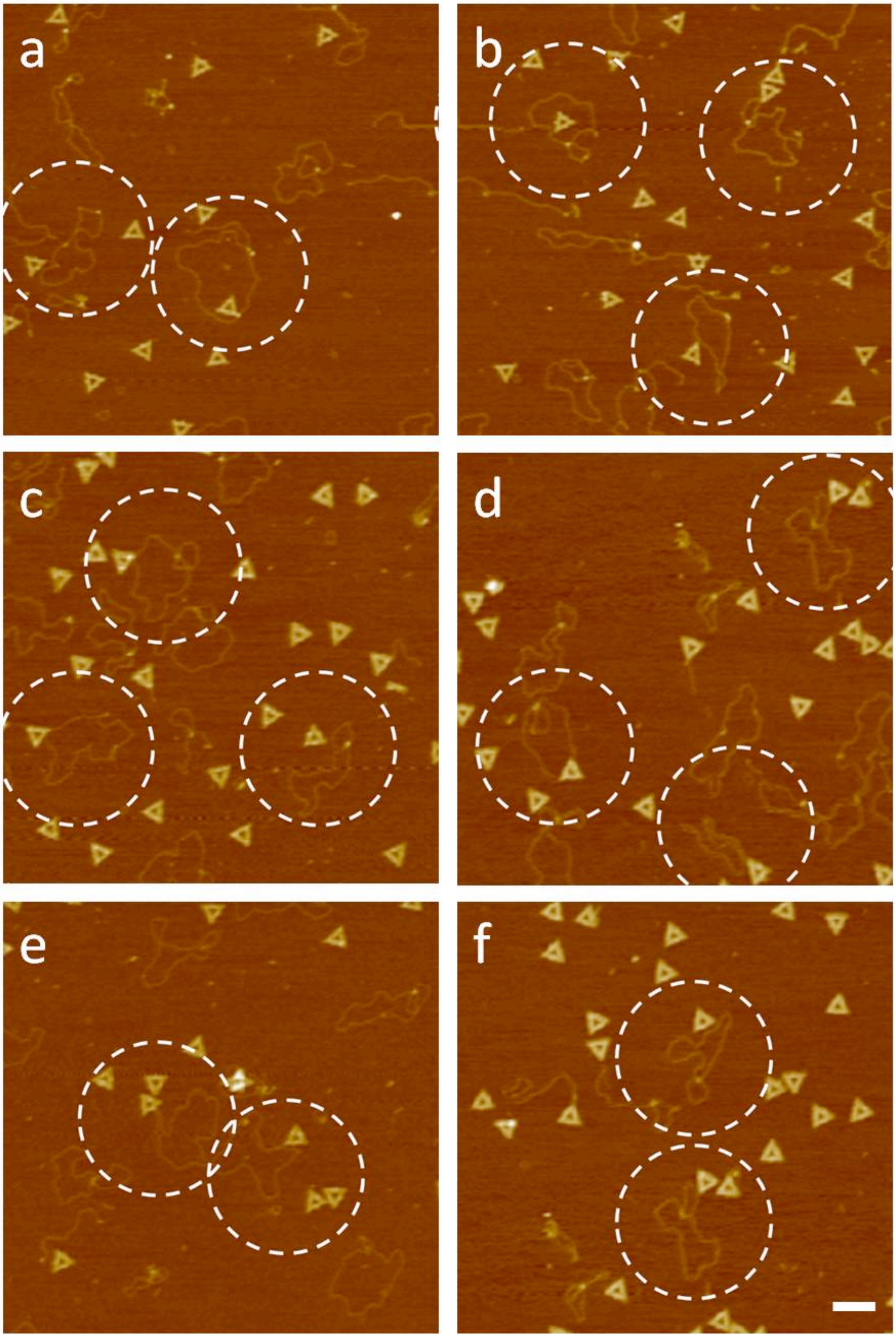

Supplementary Figure 6 | (a-f) The site-specific single labeling of phiX 174 by triangular shaped ID with one M3'strand at the edge middle of origami. Scale bar: 200 nm.

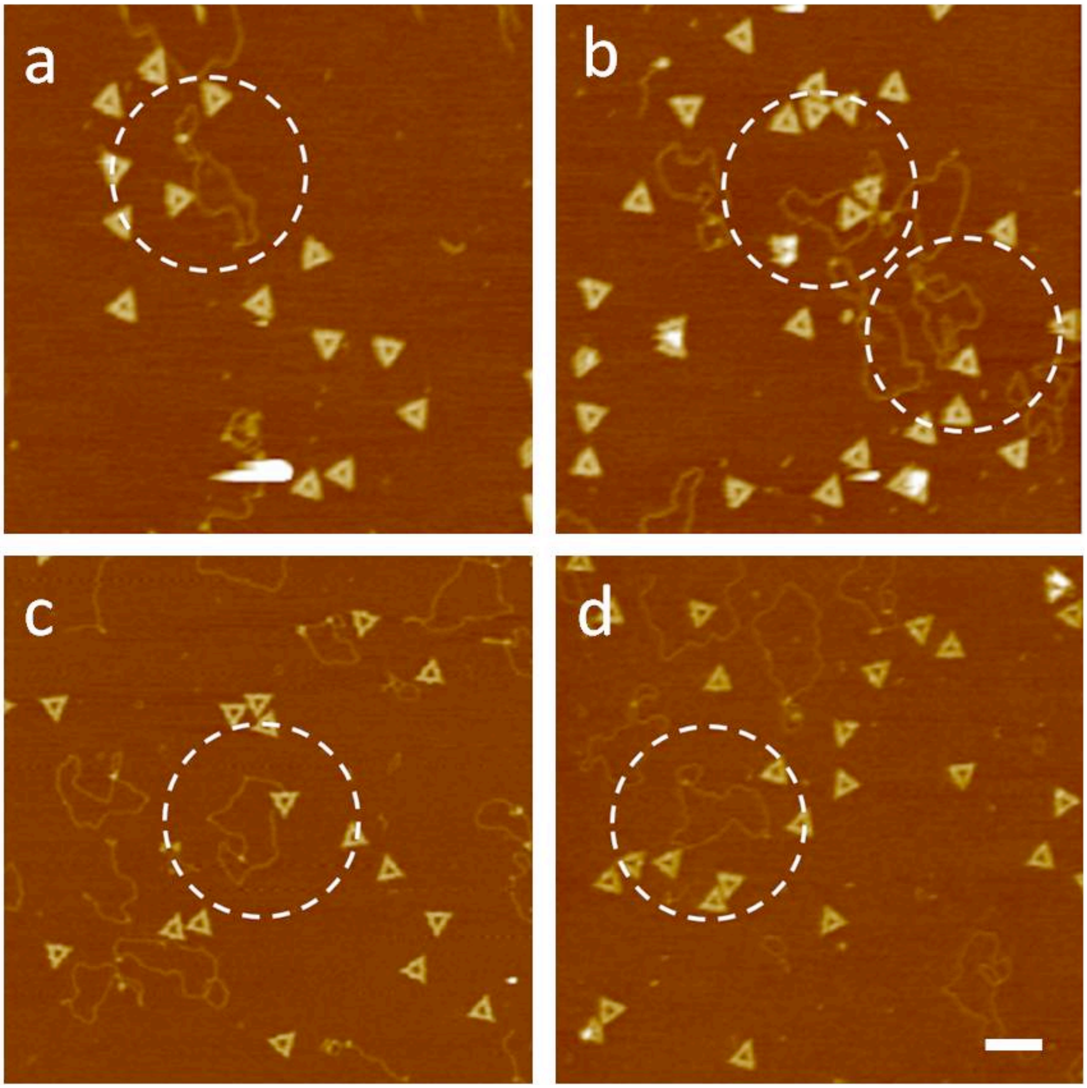

Supplementary Figure 7 | (a-d) The site-specific single labeling of phiX 174 by triangular shaped ID with one M3' strand at the inner of origami. Scale bar: 200 nm.

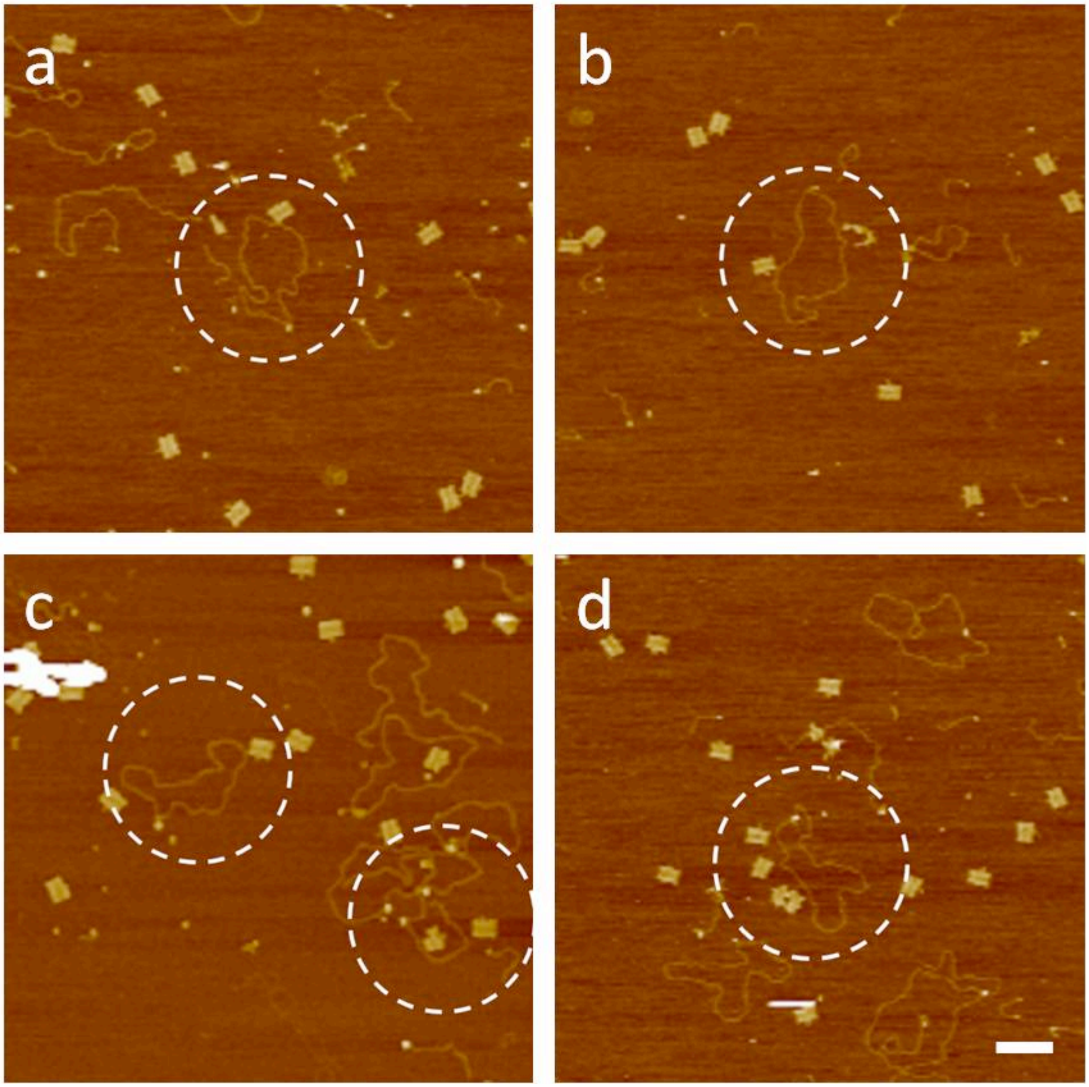

Supplementary Figure 8 | (a-d) The site-specific single labeling of phiX 174 by rectangular shaped ID with one M3' strand at the corner point of origami. Scale bar: 200 nm.

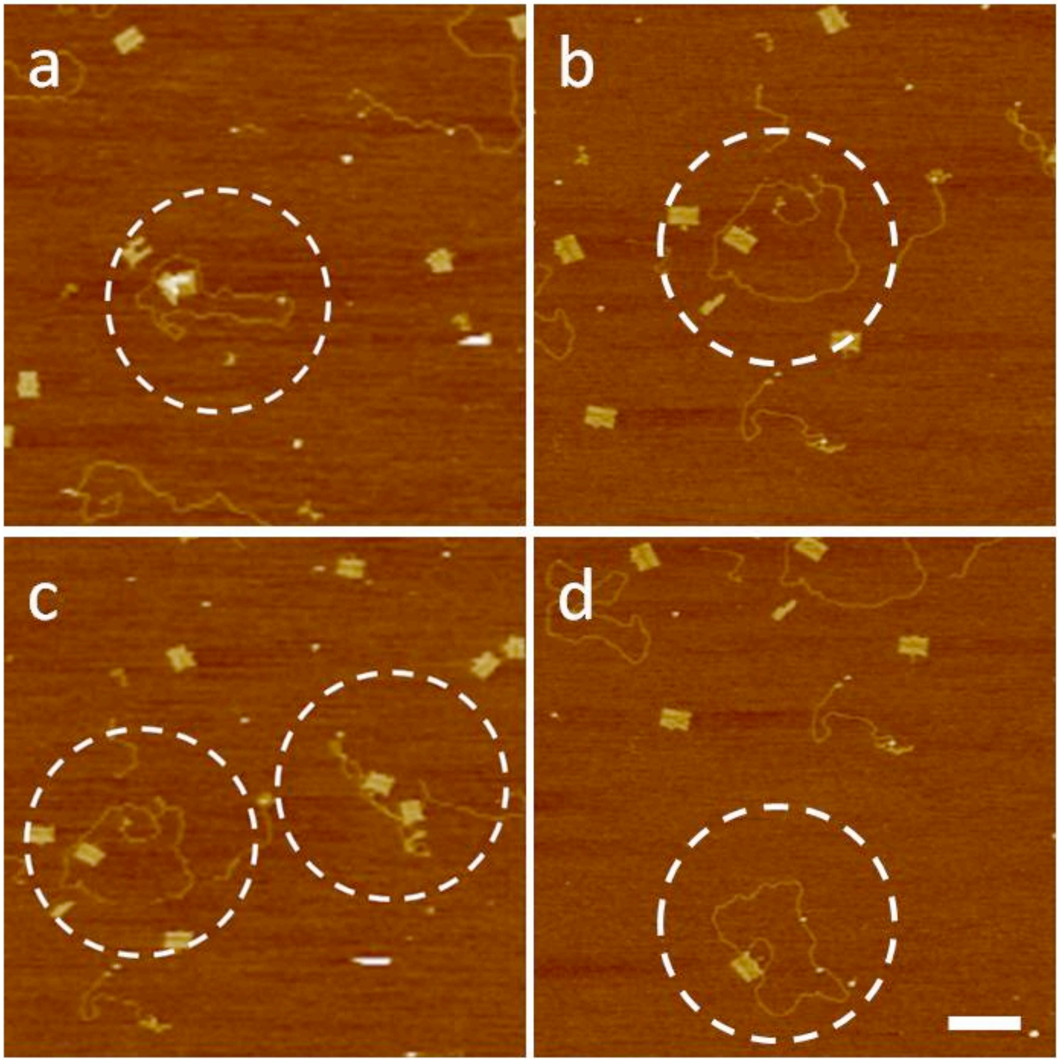

Supplementary Figure 9 | (a-d) The site-specific single labeling of phiX 174 by rectangular shaped ID with one M3'strand at the edge middle of origami. Scale bar: 200 nm.

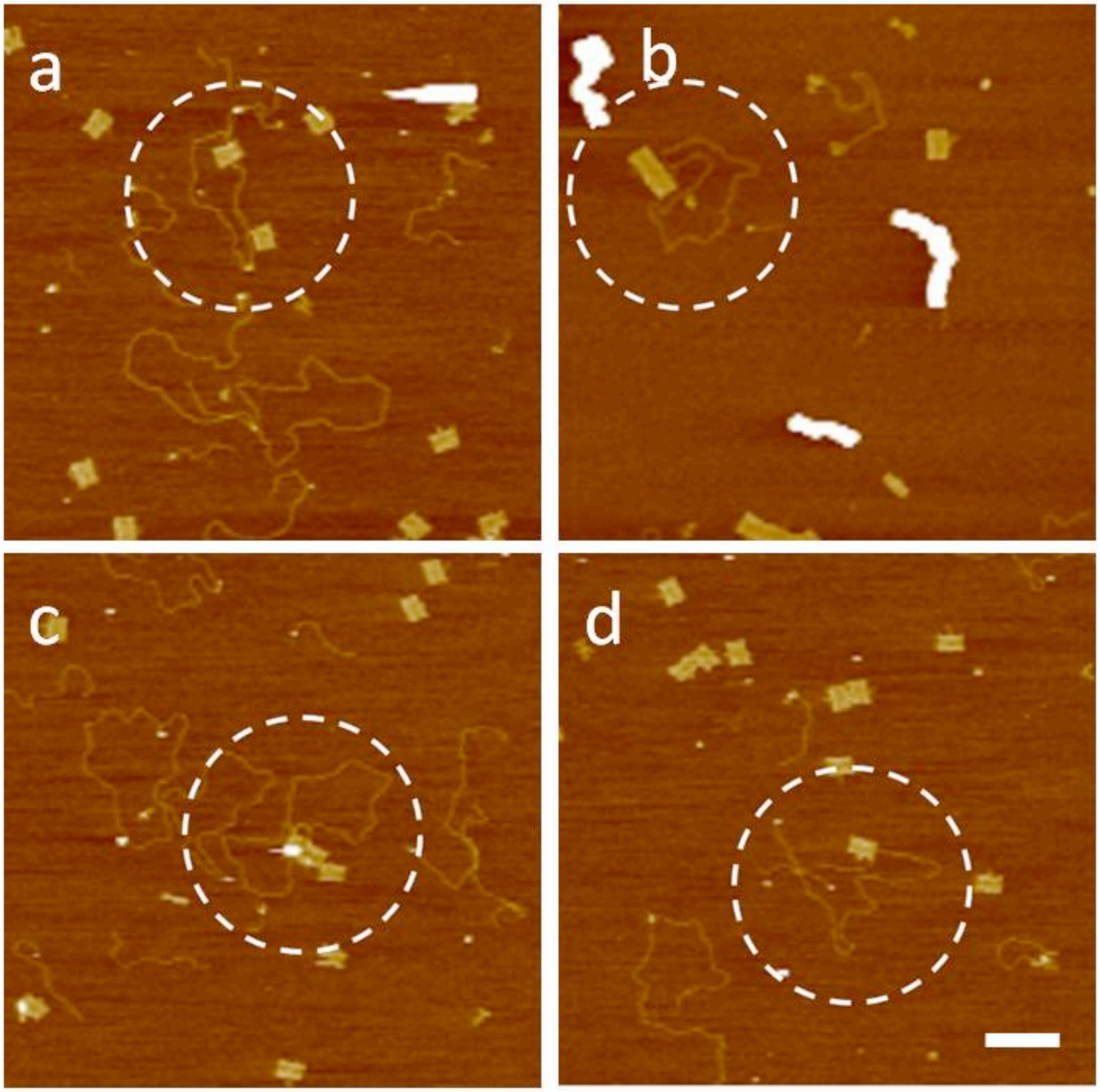

Supplementary Figure 10 | (a-d) The site-specific single labeling of phiX 174 by rectangular shaped ID with one M3'strand at the inner of origami. Scale bar: 200 nm.

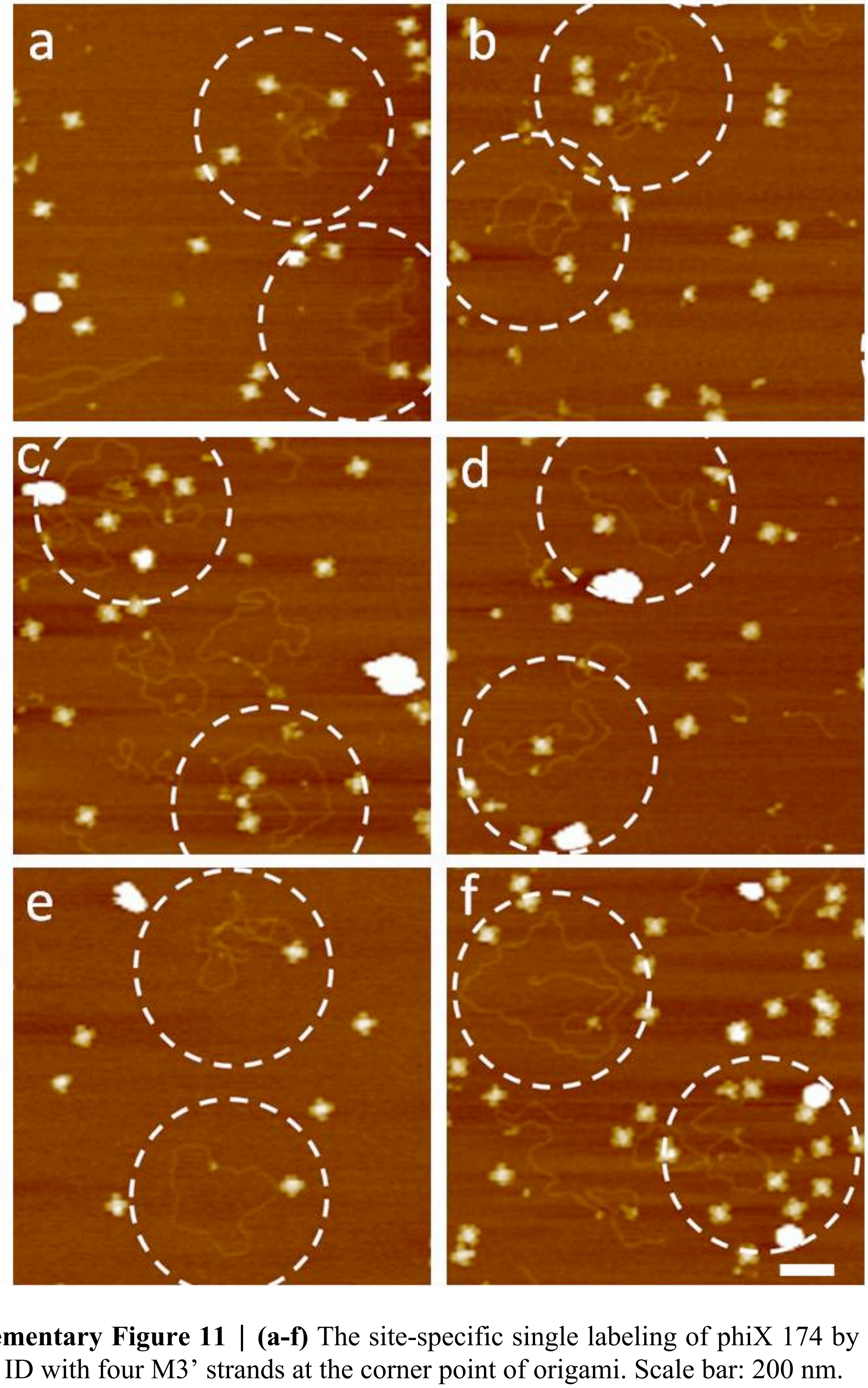

Supplementary Figure 11 | (a-f) The site-specific single labeling of phiX 174 by cross shaped ID with four M3’ strands at the corner point of origami. Scale bar: 200 nm.

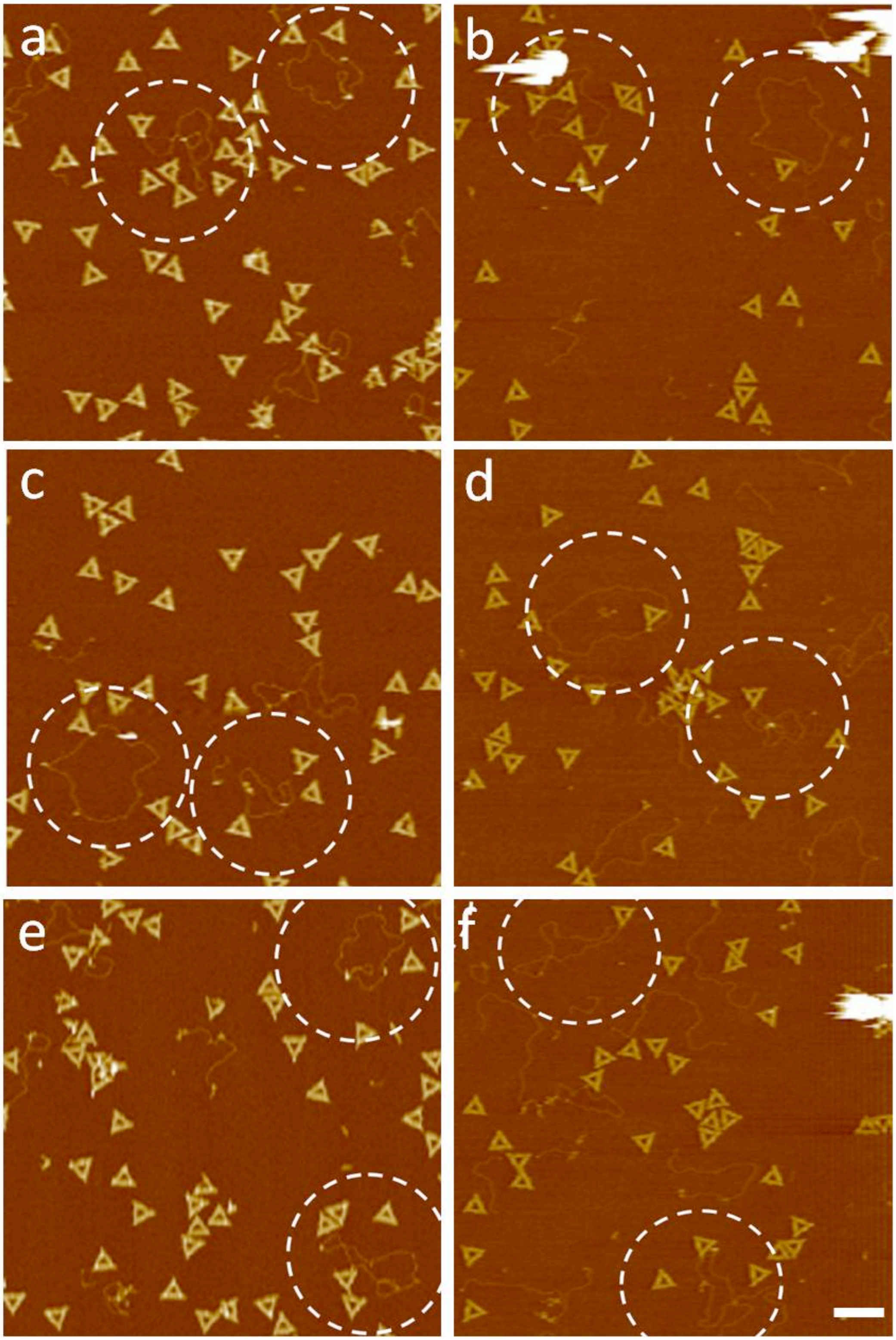

Supplementary Figure 12 | (a-f) The site-specific single labeling of phiX 174 by triangular shaped ID with three M3'strands at the corner point of origami. Scale bar: 200 nm.

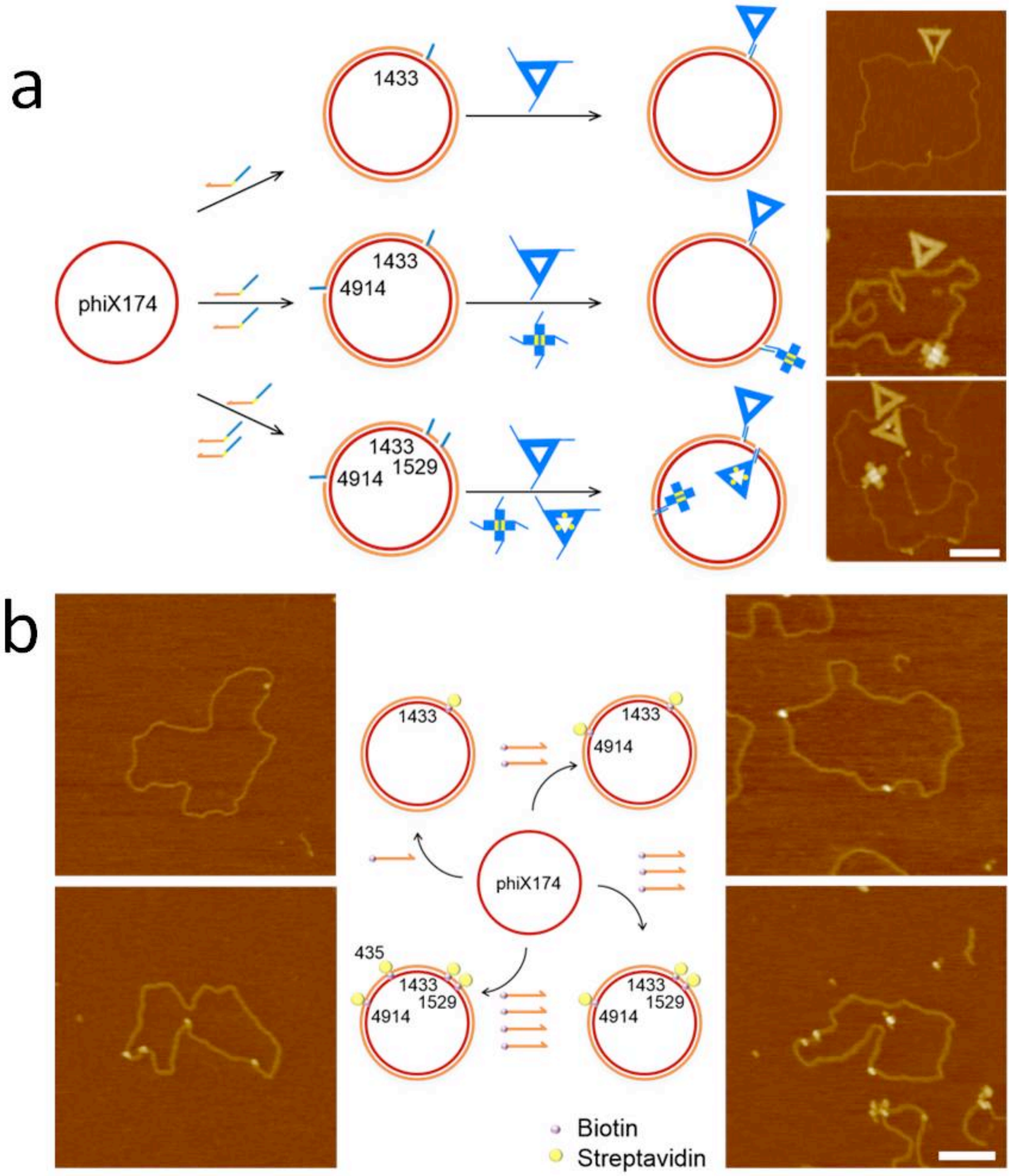

Supplementary Figure 13 | Schematic illustrations and AFM images of the processes of origami shape IDs (a) and STVs (b) for specific-labeling of phiX174. Scale bar: 200nm.

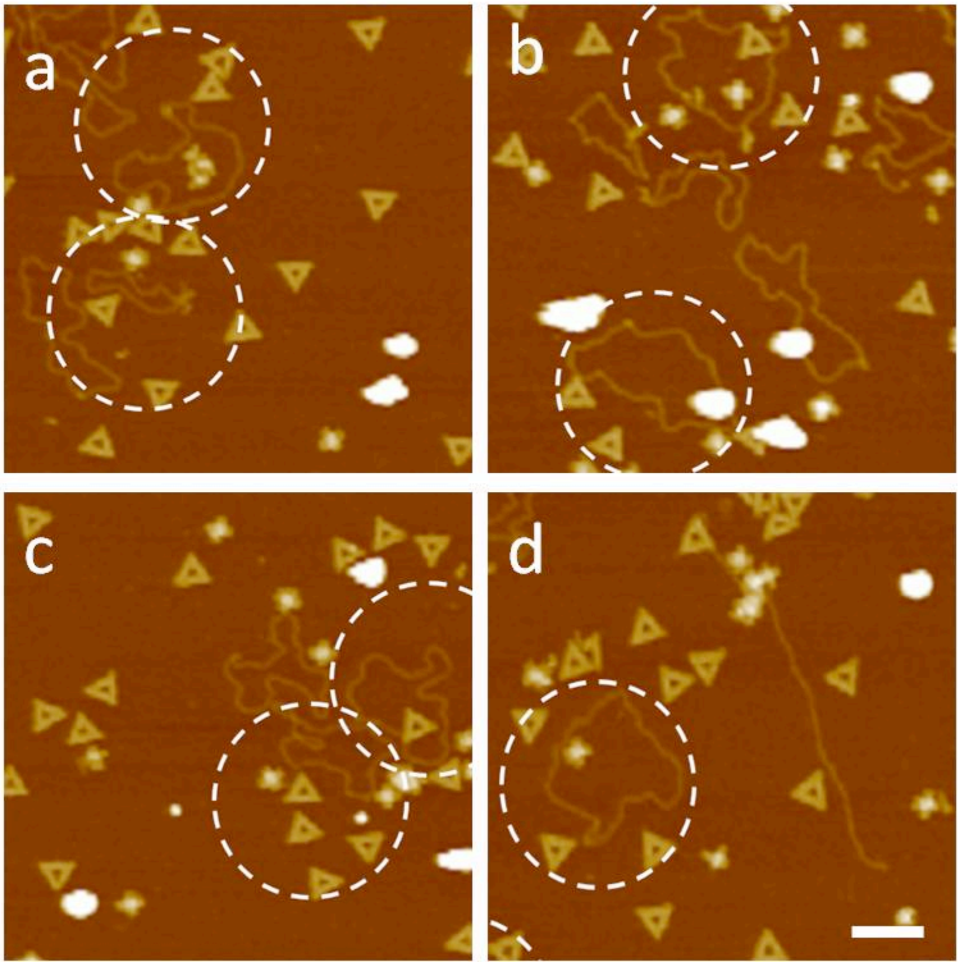

Supplementary Figure 14 | (a-d) The site-specific dual labeling of phiX 174 by cross and triangular shaped ID with M3'strands at the corner point of origami. Scale bar: 200nm.

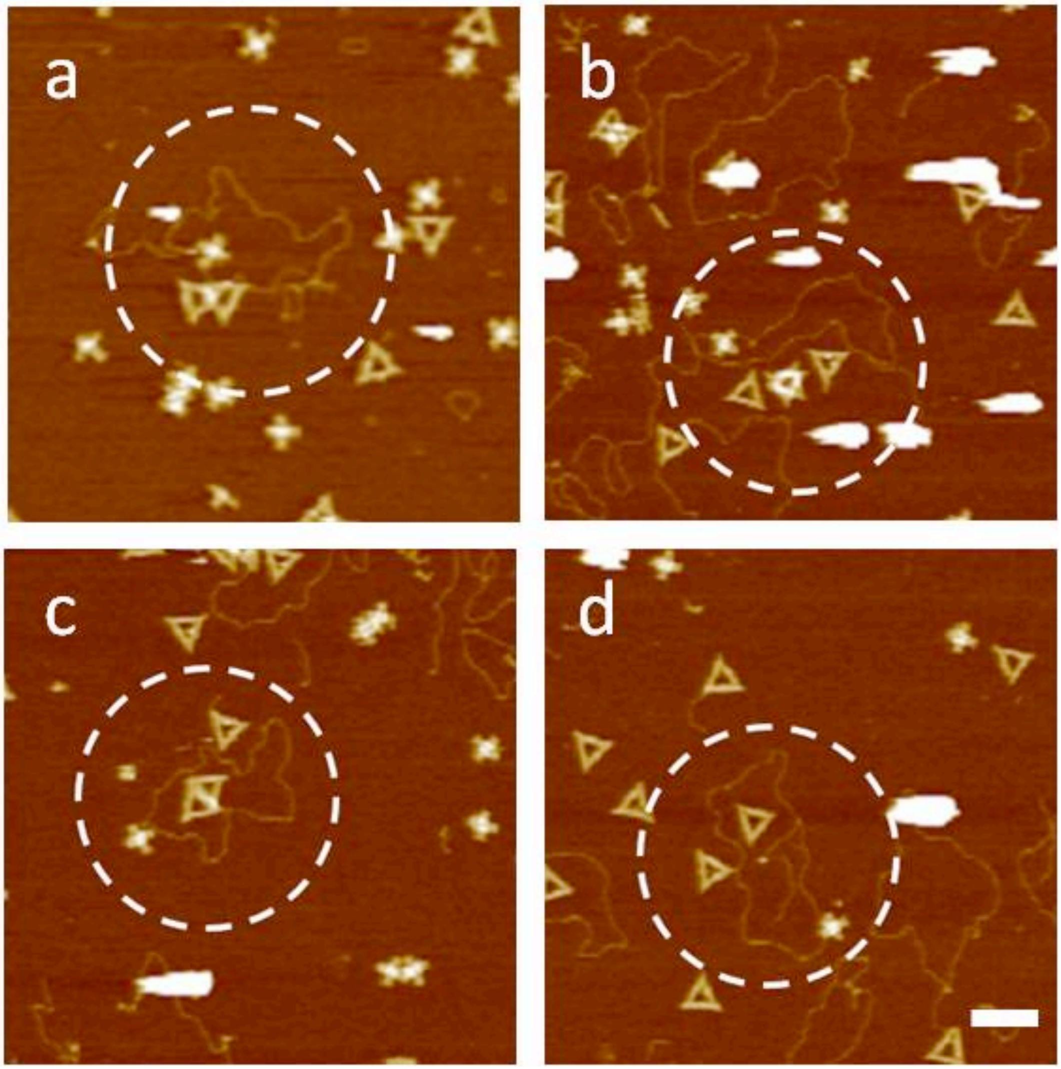

Supplementary Figure 15 | (a-d) The site-specific triple labeling of phiX 174 by cross, triangular and STV-decorated (inner edge middle) triangular shaped ID with M3'strands at the corner point of origami. Scale bar: 200nm.

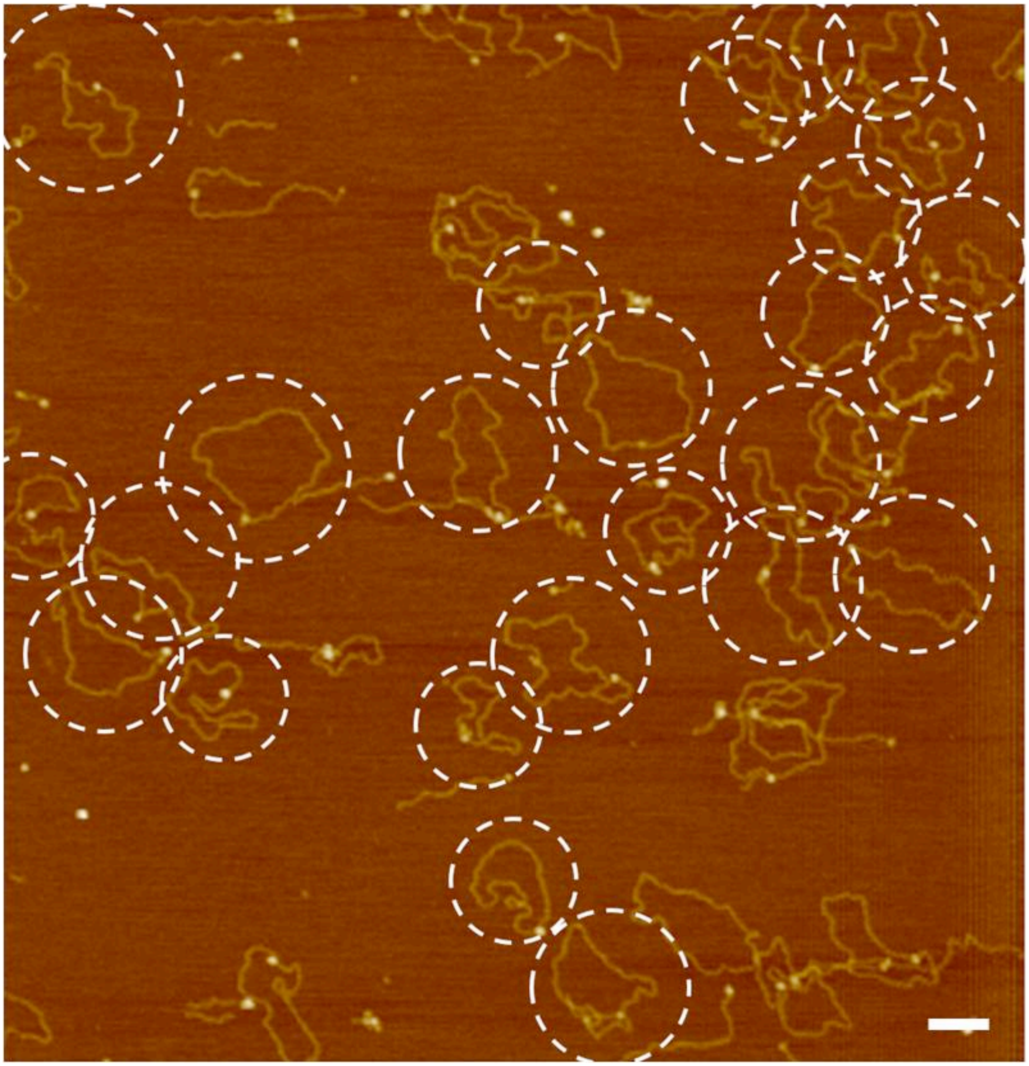

Supplementary Figure 16 | The site-specific single-site labeling of phiX174 by STV. Scale bar: 200nm.

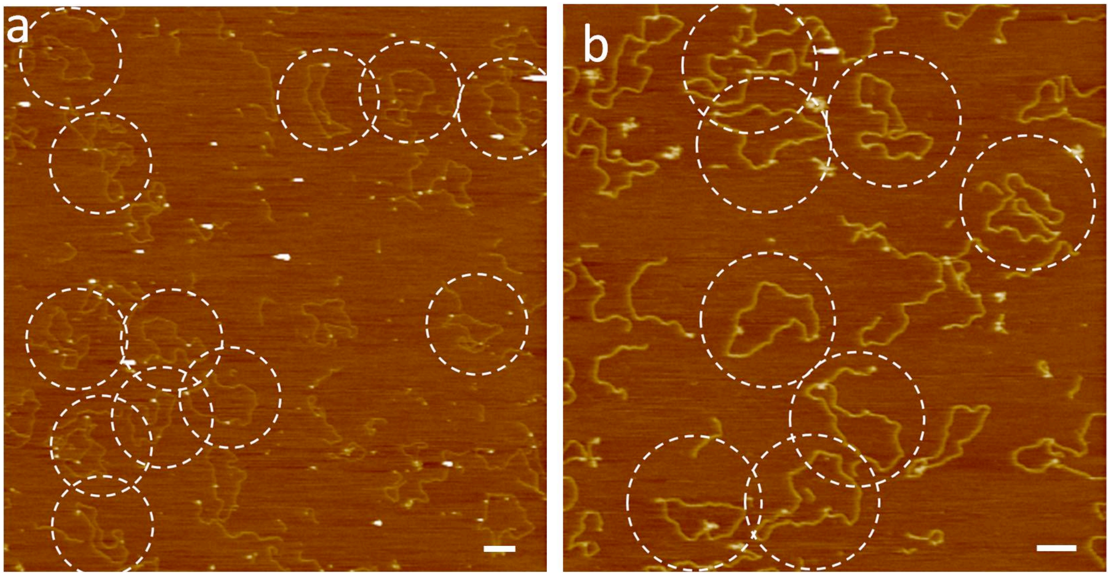

Supplementary Figure 17 | (a, b) The site-specific two-site labeling of phiX174 by STV. Scale bar: 200nm.

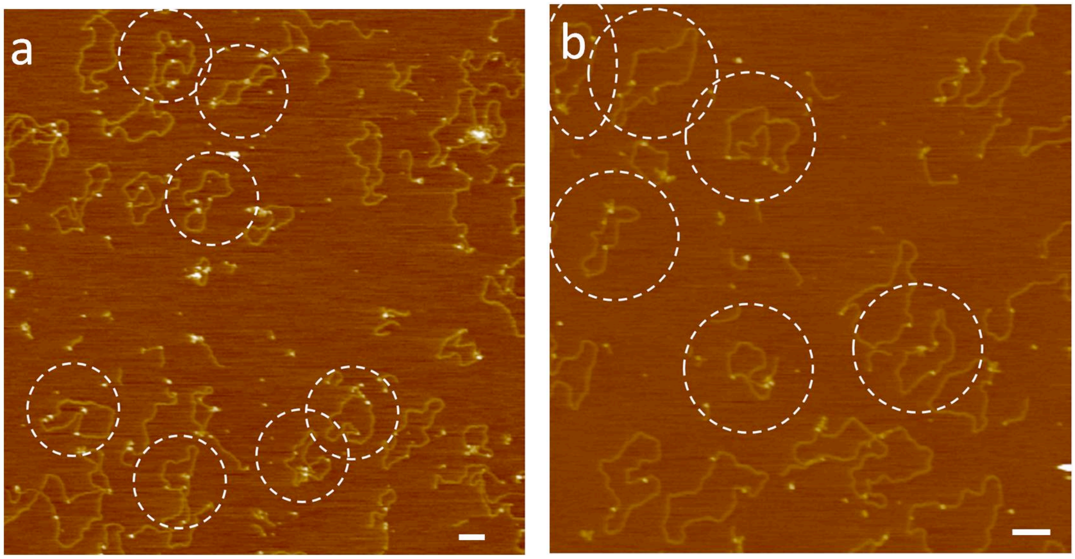

Supplementary Figure 18 | (a,b) The site-specific three-site labeling of phiX174 by STV. Scale bar: 200nm.

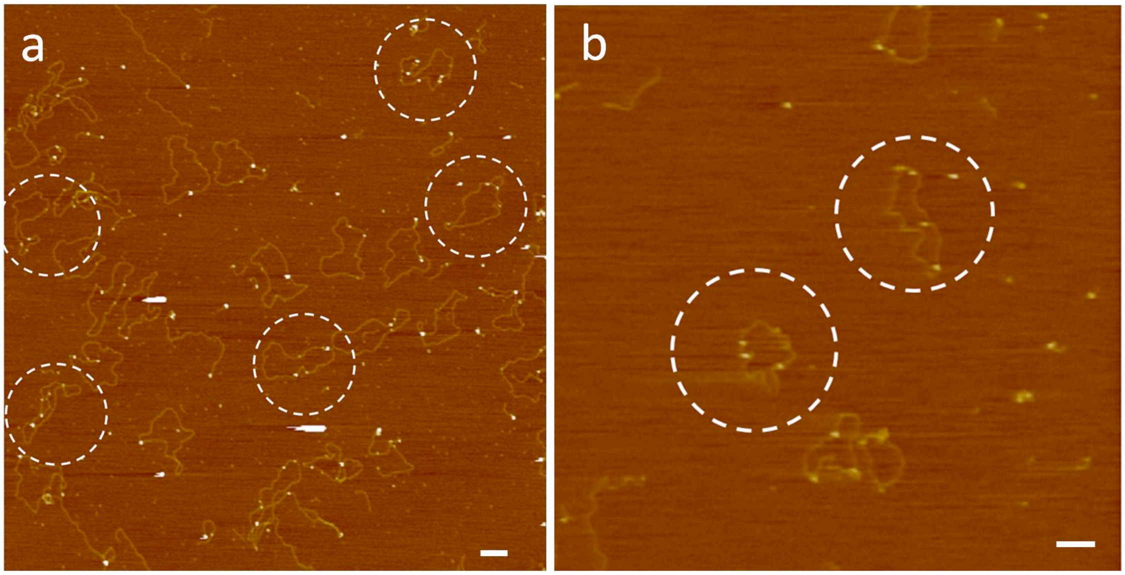

Supplementary Figure 19 | (a, b) The site-specific four-site labeling of phiX174 by STV. Scale bar: 200nm.

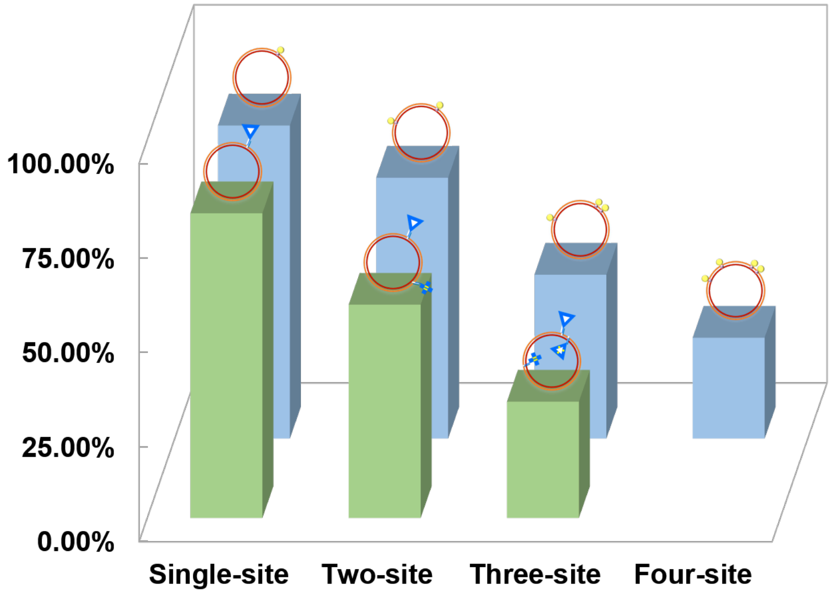

Supplementary Figure 20 | The labeling efficiency of shape IDs (green columns) and STVs (blue columns) on phiX 174 template.

The labeling efficiency is based on the binding of M1 strand to DNA template, the primers extension under DNA polymerases and the labeling of the DNA origami IDs to M3 strand. Therefore, we utilized several 20-base 5' end biotin modified primers to investigate the extension efficiency of M1 strand on DNA. The schematic illustrations (Fig. S2A) and AFM images (Fig. S16-S19) showed the closed circularity DNA labeled with STVs at distinct sites. Because the binding efficiency of STV and biotin is almost 100%, we considered STV labeling efficiency as the extension efficiency of M1 strand onphiX174 template. The statistic analysis data showed that the extension efficiency was decreased from 82.8%, 69.0%, 43.3% to 26.7% as the number of modified primers increased from one to four. Considering the extension efficiency of M1 strand, single, dual and triple labeling efficiency of shape IDs are 80.6%, 56.4% and 30.8%, respectively.

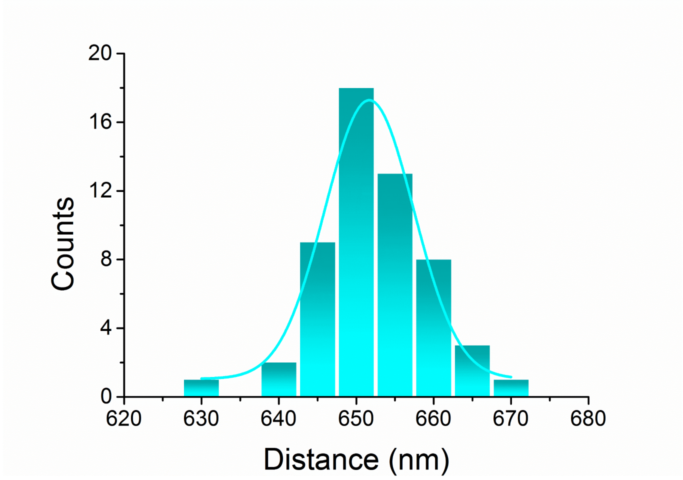

Supplementary Figure 21| Histograms for the counts of STVs as a function of distance between site 1433 and 4914 in phiX. The measured distance is 652 nm, which is in good agreement with the calculated distance 648 nm.

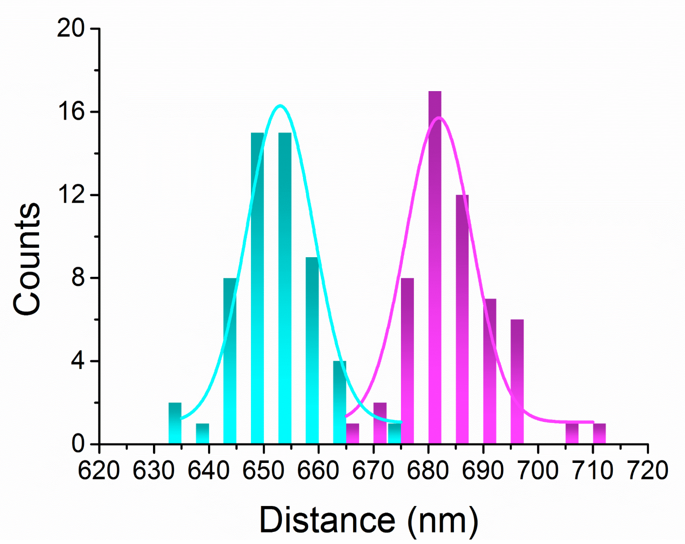

Supplementary Figure 22 | Histograms for the counts of STVs as a function of distance between site 1433, 1529 and 4914 in phiX. The measured distances are 653 nm and 682 nm, which are in good agreement with the calculated distances 648 nm and 680 nm.

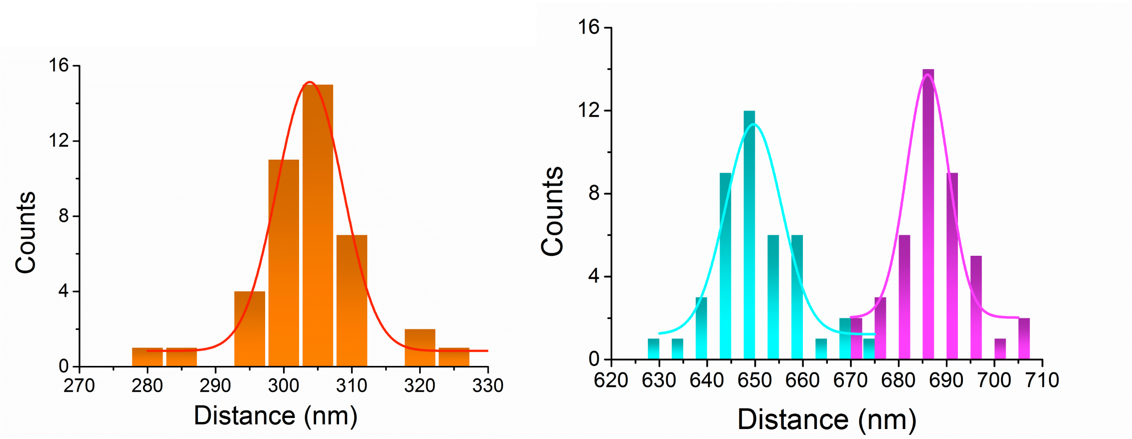

Supplementary Figure 23 | Histograms for the counts of STVs as a function of distance between site 435 (upper), 1433, 1529 (lower) and 4914 in phiX. The measured distances are 304 nm, 650 nm and 686 nm, which are in good agreement with the calculated distances 308 nm, 648 nm and 680 nm.

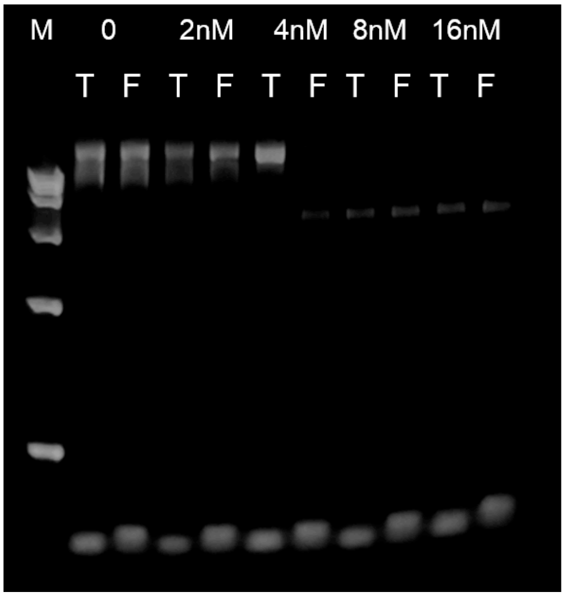

Supplementary Figure 24 | Gel electrophoresis of the phiX174 DNA template's extension by perfectly matched primer (line T) and one-base mismatched primer (line F) assisted under different concentrations of AuNP. Line M, DL15,000 maker.

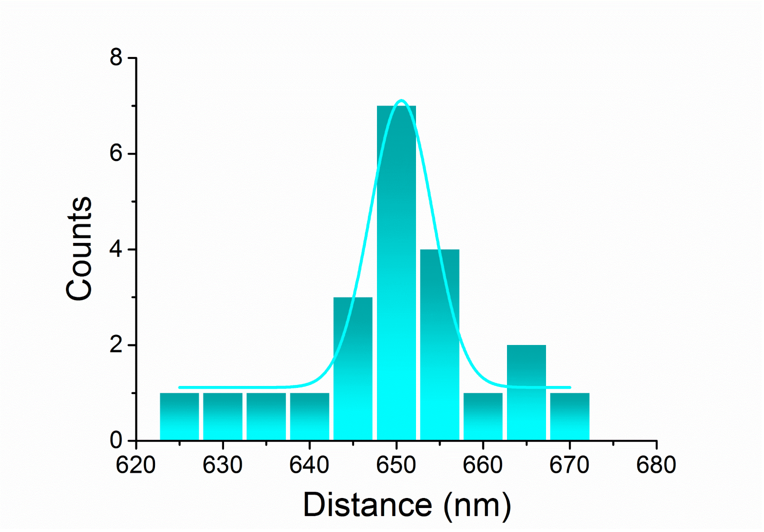

Supplementary Figure 25 | Histogram of specificity of shape IDs labeling based on AuNPs-enhanced primer extension. The distance between site 1433 and 4914 is 651 nm, which is in good agreement with the calculated distance 648 nm.

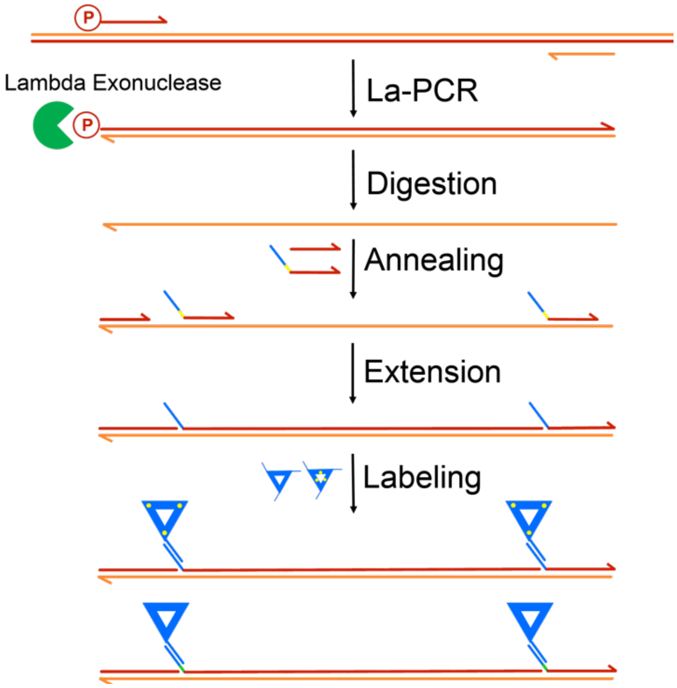

Supplementary Figure 26 | The process for labeling genomic DNA extracted from human blood samples, which is subjected to long-range PCR amplification, digestion by lambda exonuclease to produce single strand DNA, allele-specific extension and labeling with shape IDs.

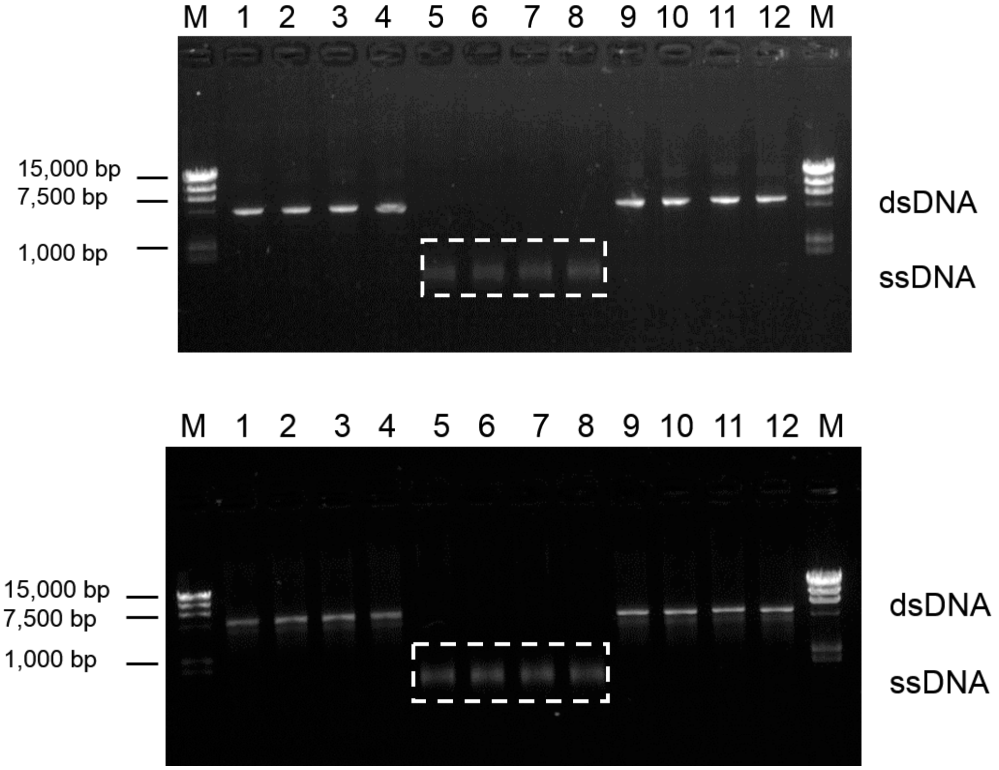

Supplementary Figure 27 | Gel electrophoresis of regenerated dsDNA from ssDNA. 4.6-kb long-range PCR products of four different AMD samples (lanes 1–4) were digested with lambda exonuclease to produce the corresponding ssDNA (lanes 5–8, respectively). After primer extension, dsDNA samples were regenerated (lanes 9–12, respectively). Line M, DL15,000 maker.

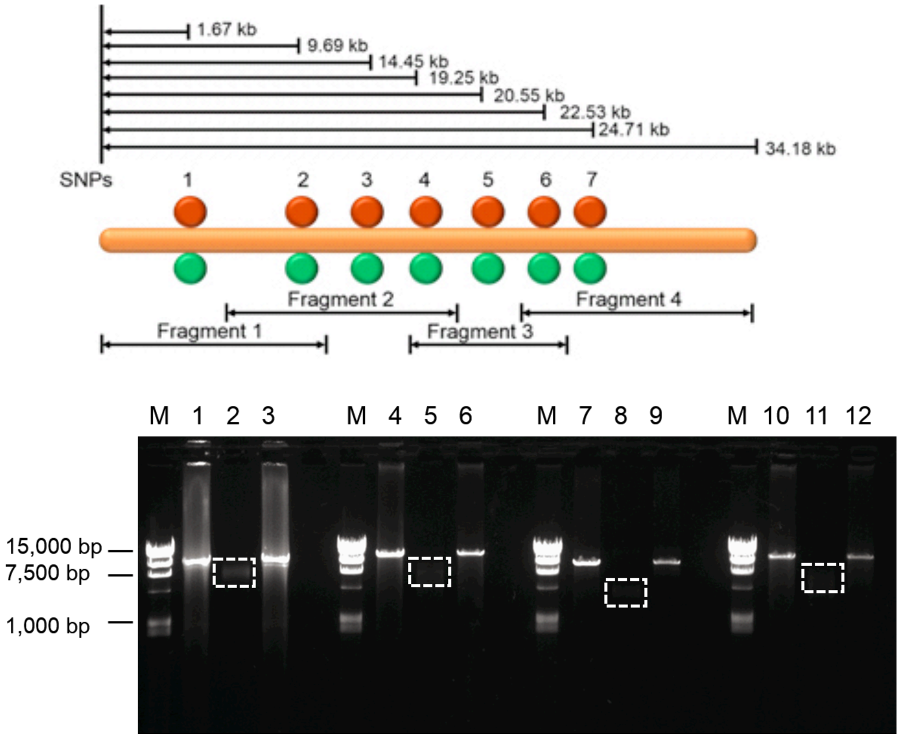

Supplementary Figure 28 | Upper: schematic showing of a 34-kb region AMD sample, which is on the chromosome 10 from the Han Chinese population. It consists of seven SNP alleles that are split into four overlapping fragments. Each fragment contains two or three SNP loci, which has at least one SNP at the ends as "joint locus". The "joint locus" is heterozygously shared by its adjacent fragment and used for subsequent fragment connection. Lower: Gel electrophoresis of regenerated dsDNA from ssDNA. Four fragments in 34-kb were generated by long-range PCR of one AMD samples (lanes 1, 4, 7 and 10) were digested with lambda exonuclease to produce the corresponding ssDNA (lanes 2, 5, 8 and 11, respectively). After primer extension, dsDNA samples were regenerated (lanes 3, 6, 9 and 12, respectively). Line M, DL15,000 maker.

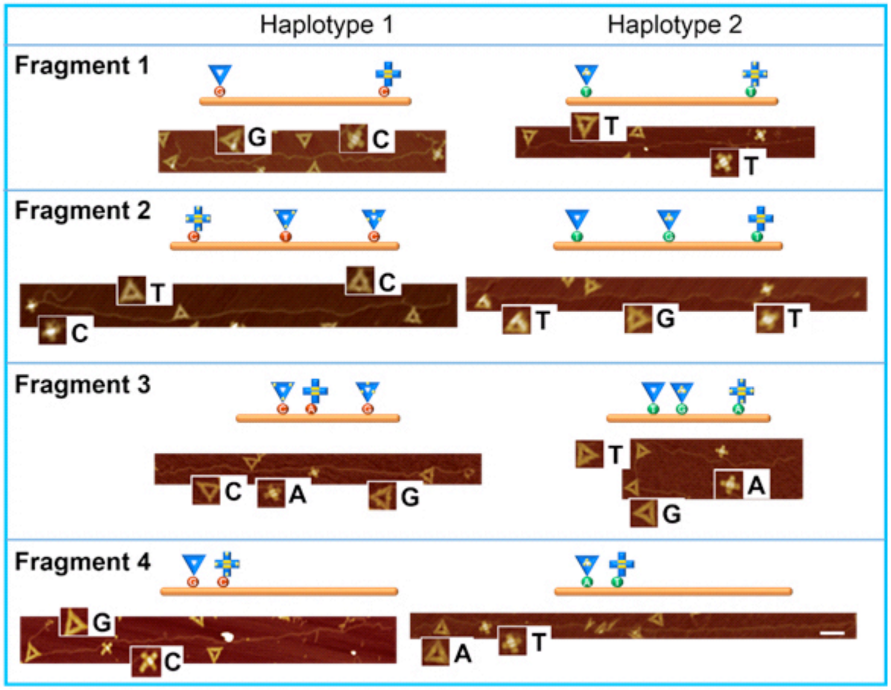

Supplementary Figure 29 | AFM images for haplotypes of four fragments. The haplotypes of each fragment can be directly read. For example, in the fragment 2, the two haplotypes of C-T-C and T-G-T correspond to SNPs 2, 3 and 4, respectively. Connection of the four fragments reconstructs continuous haplotypes for this 34-kb region. That is, haplotype 1containsG-C-T-C-A-G-C, and haplotype 2 contains T-T-G-T-G-A-T.

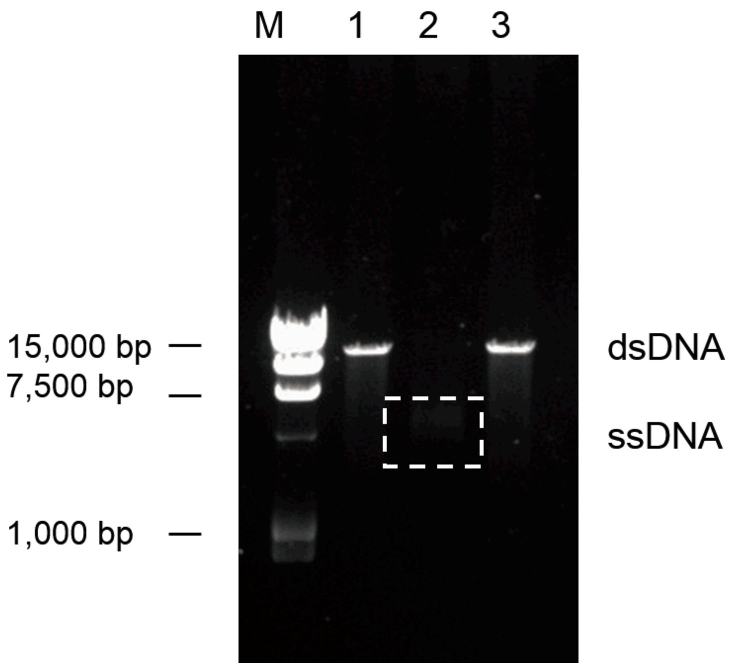

Supplementary Figure 30 | Gel electrophoresis of regenerated dsDNA from ssDNA. 12-kb long-range PCR product of one DNA samples (lanes 1) was digested with lambda exonuclease to produce the corresponding ssDNA (lanes 2). After primer extension, dsDNA samples were regenerated (lanes 3). Line M, DL15,000 maker.

For the three 4.6-kb region samples on the chromosome 3 (fig 4 in the old edition, new fig 5a), each sample from the patients was labeled six times independently, their yields were ~85%. For the 34-kb region AMD sample (fig 5a in the old edition, new fig 5b), it was labeled four times independently, the yields were 75%. For the sample of 12-kb region of p53 gene, it was labeled six times independently, the yields were 82%.

Supplementary Table 1| The labeling efficiency of shape IDs with different number of M3' strands in one origami (considering the extension efficiency of M1 strand). The black line inside the dashed circle indicates M3' strand.

**Shape IDs with M3' strands**	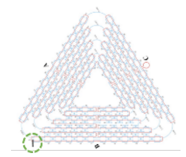	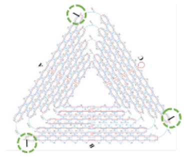	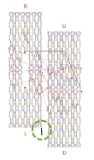	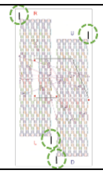
**Labeling efficiency**	**66.7%**	**80.6%**	**77.7%**	**85.0%**

Supplementary Table 2|Sequences of staple strands for STV-decorated triangular and cross shaped DNA origami IDs. Sequence TTTTT is used as a spacer region.

Staple strands		Sequences
triA1bio	3'-Biontin	CGGGGTTTCCTCAAGAGAAGGATTTTGAATTA TTTTT
triB1bio	3'-Biontin	TCATATGTGTAATCGTAAAACTAGTCATTTTC TTTTT
triC1bio	3'-Biontin	TCGGGAGATATACAGTAACAGTACAAATAATT TTTTT
triLA2Cbio	3'-Biontin	TTAATTTCATCTTAGACTTTACAA TTTTT
triLB2Abio	3'-Biontin	TTTAATTGTATTTCCACCAGAGCC TTTTT
triLC2Bbio	3'-Biontin	CGGTGCGGGCCTTCCAAAAACATT TTTTT
triA4bio	3'-Biontin	ATGGTTTATGTCACAATCAATAGATATTAAAC TTTTT
triB4bio	3'-Biontin	TTCGAGCTAAGACTTCAAATATCGGGAACGAG TTTTT
triC4bio	3'-Biontin	CGACCAGTACATTGGCAGATTCACCTGATTGC TTTTT
croM4bio	5'-Biontin	TTTTT AATAGCAATAGATGGGCGCATCGTACCGTATC
croM87bio	5'-Biontin	TTTTT AATTCATCAACCATATCAAAATTATAGATTTT
croM93bio	3'-Biontin	ATAGTCAGGGAAGCCCGAAAGACTCAATTCTG TTTTT
croM175bio	3'-Biontin	CCAGAATGAAGCGTCATACATGGCAGCCCGGA TTTTT

Supplementary Table 3| Sequences of M-strands and M3' staple strands in triangular, cross and rectangular shaped IDs for site-specific single- and multi-labeling of phiX174.

M-strands and M3' staple strands	Allele	Location	Sequences
SNP1433tri B03			GTC GCT CTC TCA AGC AGA AT TTTTT GGCAT CAAAT TTGGG GCGCG AGCTA GTTAA AG
SNP1433tri B37			GTC GCT CTC TCA AGC AGA AT TTTTT ACAGG TAGAA AGATT CATCA GTTGA GATTT AG
SNP1433tri B65			GTC GCT CTC TCA AGC AGA AT TTTTT CCTGA CGAGA AACAC CAGAA CGAGT AGGCT GCTCA TTCAG TGA
SNP1433cro M16			GTC GCT CTC TCA AGC AGA AT TTTTT TAAGA AAAGA TTGAC CGTAA TGGGC CAGCT TT
SNP1433cro AL3			GTC GCT CTC TCA AGC AGA AT TTTTT AATAA TAAGA GCAAG AGAAT TGAGT TAAGC CC
SNP1433cro AL6			GTC GCT CTC TCA AGC AGA AT TTTTT AGCTG ATTGC CCTTC ACAGT GAGAC GGGCA AC
SNP1433rec 121			GTC GCT CTC TCA AGC AGA AT TTTTT GGTCA ATAAC CTGTT TATAT CGCG
SNP1433rec 211			GTC GCT CTC TCA AGC AGA AT TTTTT CTGTA GCTCA ACATG TATTG CTGA
SNP1433rec 205			GTC GCT CTC TCA AGC AGA AT TTTTT CGATG GCCCA CTACG TAAAC CGTC
SNP1433		#1433	AT TCT GCT TGA GAG AGC GAC TTTTT TACGG TCAGG CATCC ACGGC
SNP1433tri A65			GTC GCT CTC TCA AGC AGA AT TTTTT TGCTA TTTTG CACCC AGCTA CAATT TTGTT TTGAA GCCTT AAA
SNP1433tri B65			GTC GCT CTC TCA AGC AGA AT TTTTT CCTGA CGAGA AACAC CAGAA CGAGT AGGCT GCTCA TTCAG TGA
SNP1433tri C65			GTC GCT CTC TCA AGC AGA AT TTTTT ACGTG GACTC CAACG TCAAA GGGCG AATTT GGAAC AAGAG TCC
SNP1433		#1433	AT TCT GCT TGA GAG AGC GAC TTTTT TACGG TCAGG CATCC ACGGC
SNP1529tri A65			GAA TGT CCG CGT CAG TCG TC TTTTT TGCTA TTTTG CACCC AGCTA CAATT TTGTT TTGAA GCCTT AAA
SNP1529tri B65			GAA TGT CCG CGT CAG TCG TC TTTTT CCTGA CGAGA AACAC CAGAA CGAGT AGGCT GCTCA

			TTCAG TGA
SNP1529tri C65			GAA TGT CCG CGT CAG TCG TC TTTTT ACGTG GACTC CAACG TCAAA GGGCG AATTT GGAAC AAGAG TCC
SNP1529		#1529	GA CGA CTG ACG CGG ACA TTC TTTTT TCAGG AGGAA GCGGA GCAGT
SNP4914cro AU6			GTG CTG ACA CGG CCT GAT CC TTTTT GAGGA CTAAA GACTT TCGGC TACAG AGGCT TT
SNP4914cro AD1			GTG CTG ACA CGG CCT GAT CC TTTTT CGTTA ATATT TTGTT AATAT TTAAA TTGTA AA
SNP4914cro AL6			GTG CTG ACA CGG CCT GAT CC TTTTT AGCTG ATTGC CCTTC ACAGT GAGAC GGGCA AC
SNP4914cro AR1			GTG CTG ACA CGG CCT GAT CC TTTTT GTTAA ATAAG AATAA AGTGT GATAA ATAAG GC
SNP4914	G	#4914	GG ATC AGG CCG TGT CAG CAC TTTTT TGTAG CGAAC TGCGA TGGGC
SNP4914C	C	#4914	AT TCT GCT TGA GAG AGC GAC TTTTT TGTAG CGAAC TGCGA TGGGG
SNP1463cro AU6			GTG CTG ACA CGG CCT GAT CC TTTTT GAGGA CTAAA GACTT TCGGC TACAG AGGCT TT
SNP1463cro AD1			GTG CTG ACA CGG CCT GAT CC TTTTT CGTTA ATATT TTGTT AATAT TTAAA TTGTA AA
SNP1463cro AL6			GTG CTG ACA CGG CCT GAT CC TTTTT AGCTG ATTGC CCTTC ACAGT GAGAC GGGCA AC
SNP1463cro AR1			GTG CTG ACA CGG CCT GAT CC TTTTT GTTAA ATAAG AATAA AGTGT GATAA ATAAG GC
SNP1463		#1463	GG ATC AGG CCG TGT CAG CAC TTTTT CTTGA TTAAG CTCAT TAGGG

Supplementary Table 4| Sequences of strands for site-specific STV-labeling of phiX174.

Strands		Location	Sequences
SNP435bio	5'-Biontin	#435	TTTTT GCCAA AGCGG TCTGG AAACG
SNP1433bio	5'-Biontin	#1433	TTTTT TACGGTCAGGCATCCACGGC
SNP1529bio	5'-Biontin	#1529	TTTTT TCAGG AGGAA GCGGA GCAGT
SNP4914bio	5'-Biontin	#4914	TTTTT TGTAGCGAACTGCGATGGGC

Supplementary Table 5| Sequences of PCR primers, M-strands and M3' staple strands in shape IDs for site-specific labeling of polymorphic sites on 4.6-kb region AMD samples (chr3:39273966-39278556).

Strands		Allete	Location	Sequences
Pri4L	5' Phosphorylated		0	TCTGCAGGCAGATAATGAGG
Pri4R			4.6kb	GAAGGGAGGGGAGAAGAAGA
SNP1-C	rs17038640	C	1.2kb	AT TCT GCT TGA GAG AGC GAC TTTTT ACAGT TGATT GGACT TTCA G
SNP1-T	rs17038640	T	1.2kb	GG ATC AGG CCG TGT CAG CAC TTTTT ACAGT TGATT GGACT TTCA A
SNP1triA 65-C				GTC GCT CTC TCA AGC AGA AT TTTTT TGCTA TTTTG CACCC AGCTA CAATT TTGTT TTGAA GCCTT AAA
SNP1triB 65-C				GTC GCT CTC TCA AGC AGA AT TTTTT CCTGA CGAGA AACAC CAGAA CGAGT AGGCT GCTCA TTCAG TGA
SNP1triC 65-C				GTC GCT CTC TCA AGC AGA AT TTTTT ACGTG GACTC CAACG TCAAA GGGCG AATTT GGAAC AAGAG TCC
SNP1triA 65-T				GTG CTG ACA CGG CCT GAT CC TTTTT TGCTA TTTTG CACCC AGCTA CAATT TTGTT TTGAA GCCTT AAA
SNP1triB 65-T				GTG CTG ACA CGG CCT GAT CC TTTTT CCTGA CGAGA AACAC CAGAA CGAGT AGGCT GCTCA TTCAG TGA
SNP1triC 65-T				GTG CTG ACA CGG CCT GAT CC TTTTT ACGTG GACTC CAACG TCAAA GGGCG AATTT GGAAC AAGAG TCC
SNP2-C	rs4676487	C	4.2kb	AT TCT GCT TGA GAG AGC GAC TTTTT CCTTC CCAGG GACCT TGCC G
SNP2-T	rs4676487	T	4.2kb	GG ATC AGG CCG TGT CAG CAC TTTTT CCTTC CCAGG GACCT TGCC A
SNP2triA 65-C				GTG CTG ACA CGG CCT GAT CC TTTTT TGCTA TTTTG CACCC AGCTA CAATT TTGTT TTGAA GCCTT AAA
SNP2triB 65-C				GTG CTG ACA CGG CCT GAT CC TTTTT CCTGA CGAGA AACAC CAGAA CGAGT AGGCT GCTCA TTCAG TGA
SNP2triC 65-C				GTG CTG ACA CGG CCT GAT CC TTTTT ACGTG GACTC CAACG TCAAA GGGCG AATTT GGAAC AAGAG TCC

SNP2triA 65-T				GTC GCT CTC TCA AGC AGA AT TTTTT TGCTA TTTTG CACCC AGCTA CAATT TTGTT TTGAA GCCTT AAA
SNP2triB 65-T				GTC GCT CTC TCA AGC AGA AT TTTTT CCTGA CGAGA AACAC CAGAA CGAGT AGGCT GCTCA TTCAG TGA
SNP2triC 65-T				GTC GCT CTC TCA AGC AGA AT TTTTT ACGTG GACTC CAACG TCAAA GGGCG AATTT GGAAC AAGAG TCC

Supplementary Table 6| Sequences of PCR primers, M-strands and M3' staple strands in shape IDs for site-specific labeling of polymorphic sites on 34-kb region AMD samples (chr10:124200000-124235605).

Strands		Allete	Location	Sequences
Pri-1L	5' Phosphorylated		0.5kb	CCCTAAGGCAGTGGTTCT
Pri-1R			11.3kb	TGTTGGCTGGACTCGGTA
Pri-2L	5' Phosphorylated		8.6kb	TTTGGTGGTTAGTGGTTT
Pri-2R			20.3kb	CCAAGTGCGTGATTATTT
Pri-3L	5' Phosphorylated		16.9kb	GTTAGTGGCTACCCTCTTG
Pri-3R			25.3kb	GCCTTCCTACAGTCCCTC
Pri-4L	5' Phosphorylated		22.0kb	AGAAGTCATTTCGGTTGC
Pri-4R			34.2kb	CTCTTGATAAGCGTCTGG
SNP1666-G	rs2292627	G	1.7kb	GA CTG CCT CTC ACC CAC CAT TTTTT TTACC TTCCA TGACT CACT G
SNP1666-T	rs2292627	T	1.7kb	AC GTT ACC GGA CCT TAG CGA TTTTT TTACC TTCCA TGACT CACT T
SNP1666triA65-G				ATG GTG GGT GAG AGG CAG TC TTTTT TGCTA TTTTG CACCC AGCTA CAATT TTGTT TTGAA GCCTT AAA
SNP1666triB65-G				ATG GTG GGT GAG AGG CAG TC TTTTT CCTGA CGAGA AACAC CAGAA CGAGT AGGCT GCTCA TTCAG TGA
SNP1666triC65-G				ATG GTG GGT GAG AGG CAG TC TTTTT ACGTG GACTC CAACG TCAAA GGGCG AATTT GGAAC AAGAG TCC
SNP1666triA65-T				TCG CTA AGG TCC GGT AAC GT TTTTT TGCTA TTTTG CACCC AGCTA CAATT

				TTGTT TTGAA GCCTT AAA
SNP1666triB65-T				TCG CTA AGG TCC GGT AAC GT TTTTT CCTGA CGAGA AACAC CAGAA CGAGT AGGCT GCTCA TTCAG TGA
SNP1666triC65-T				TCG CTA AGG TCC GGT AAC GT TTTTT ACGTG GACTC CAACG TCAAA GGGCG AATTT GGAAC AAGAG TCC
SNP9685-C	rs11200630	C	9.7kb	AT TCT GCT TGA GAG AGC GAC TTTTT GTCCA GGCAG TAGCT TGAA C
SNP9685-T	rs11200630	T	9.7kb	GG ATC AGG CCG TGT CAG CAC TTTTT GTCCA GGCAG TAGCT TGAA T
SNP9685 croAU6-C				GTC GCT CTC TCA AGC AGA AT TTTTT GAGGA CTAAA GACTT TCGGC TACAG AGGCT TT
SNP9685 croAD1-C				GTC GCT CTC TCA AGC AGA AT TTTTT CGTTA ATATT TTGTT AATAT TTAAA TTGTA AA
SNP9685 croAL6-C				GTC GCT CTC TCA AGC AGA AT TTTTT AGCTG ATTGC CCTTC ACAGT GAGAC GGGCA AC
SNP9685 croAR1-C				GTC GCT CTC TCA AGC AGA AT TTTTT GTTAA ATAAG AATAA AGTGT GATAA ATAAG GC
SNP9685 croAU6-T				GTG CTG ACA CGG CCT GAT CC TTTTT GAGGA CTAAA GACTT TCGGC TACAG AGGCT TT
SNP9685 croAD1-T				GTG CTG ACA CGG CCT GAT CC TTTTT CGTTA ATATT TTGTT AATAT TTAAA TTGTA AA
SNP9685 croAL6-T				GTG CTG ACA CGG CCT GAT CC TTTTT AGCTG ATTGC CCTTC ACAGT GAGAC GGGCA AC
SNP9685 croAR1-T				GTG CTG ACA CGG CCT GAT CC TTTTT GTTAA ATAAG AATAA AGTGT GATAA ATAAG GC
SNP9685triA65-T				GTG CTG ACA CGG CCT GAT CC TTTTT TGCTA TTTTG CACCC AGCTA CAATT TTGTT TTGAA GCCTT AAA
SNP9685triB65-T				GTG CTG ACA CGG CCT GAT CC TTTTT CCTGA CGAGA AACAC CAGAA CGAGT AGGCT GCTCA TTCAG TGA
SNP9685triC65-T				GTG CTG ACA CGG CCT GAT CC TTTTT ACGTG GACTC CAACG TCAAA GGGCG AATTT GGAAC AAGAG TCC

SNP14449-G	rs10490924	G	14.4kb	GA CGA CTG ACG CGG ACA TTC TTTTT ACACT CCATG ATCCC AGCT G
SNP14449-T	rs10490924	T	14.4kb	CT AAC CTT GTG TCG TAG ACA TTTTT ACACT CCATG ATCCC AGCT T
SNP14449triA65-G				GAA TGT CCG CGT CAG TCG TC TTTTT TGCTA TTTTG CACCC AGCTA CAATT TTGTT TTGAA GCCTT AAA
SNP14449triB65-G				GAA TGT CCG CGT CAG TCG TC TTTTT CCTGA CGAGA AACAC CAGAA CGAGT AGGCT GCTCA TTCAG TGA
SNP14449triC65-G				GAA TGT CCG CGT CAG TCG TC TTTTT ACGTG GACTC CAACG TCAAA GGGCG AATTT GGAAC AAGAG TCC
SNP14449triA65-T				TGT CTA CGA CAC AAG GTT AG TTTTT TGCTA TTTTG CACCC AGCTA CAATT TTGTT TTGAA GCCTT AAA
SNP14449triB65-T				TGT CTA CGA CAC AAG GTT AG TTTTT CCTGA CGAGA AACAC CAGAA CGAGT AGGCT GCTCA TTCAG TGA
SNP14449triC65-T				TGT CTA CGA CAC AAG GTT AG TTTTT ACGTG GACTC CAACG TCAAA GGGCG AATTT GGAAC AAGAG TCC
SNP19253-G	rs3829198	G	19.3kb	GA CTG CCT CTC ACC CAC CAT TTTTT GCAAA GCAGT GGGGA AGTT G
SNP19253-A	rs3829198	A	19.3kb	AC GTT ACC GGA CCT TAG CGA TTTTT GCAAA GCAGT GGGGA AGTT A
SNP19253triA65-G				ATG GTG GGT GAG AGG CAG TC TTTTT TGCTA TTTTG CACCC AGCTA CAATT TTGTT TTGAA GCCTT AAA
SNP19253triB65-G				ATG GTG GGT GAG AGG CAG TC TTTTT CCTGA CGAGA AACAC CAGAA CGAGT AGGCT GCTCA TTCAG TGA
SNP19253triC65-G				ATG GTG GGT GAG AGG CAG TC TTTTT ACGTG GACTC CAACG TCAAA GGGCG AATTT GGAAC AAGAG TCC
SNP19253triA65-A				TCG CTA AGG TCC GGT AAC GT TTTTT TGCTA TTTTG CACCC AGCTA CAATT TTGTT TTGAA GCCTT AAA
SNP19253triB65-A				TCG CTA AGG TCC GGT AAC GT TTTTT CCTGA CGAGA AACAC CAGAA CGAGT AGGCT GCTCA TTCAG TGA
SNP19253triC65-A				TCG CTA AGG TCC GGT AAC GT TTTTT ACGTG GACTC CAACG TCAAA GGGCG AATTT GGAAC AAGAG TCC
SNP1925				TCG CTA AGG TCC GGT AAC GT TTTTT

3croAU6-A				GAGGA CTAAA GACTT TCGGC TACAG AGGCT TT
SNP1925 3croAD1-A				TCG CTA AGG TCC GGT AAC GT TTTTT CGTTA ATATT TTGTT AATAT TTAAA TTGTA AA
SNP1925 3croAL6-A				TCG CTA AGG TCC GGT AAC GT TTTTT AGCTG ATTGC CCTTC ACAGT GAGAC GGGCA AC
SNP1925 3croAR1-A				TCG CTA AGG TCC GGT AAC GT TTTTT GTTAA ATAAG AATAA AGTGT GATAA ATAAG GC
SNP20545-A	rs11200638	A	20.5kb	AT TCT GCT TGA GAG AGC GAC TTTTT GCGGA CGCTG CCTTC GTCC A
SNP20545-G	rs11200638	G	20.5kb	GG ATC AGG CCG TGT CAG CAC TTTTT GCGGA CGCTG CCTTC GTCC G
SNP20545croAU6-A				GTC GCT CTC TCA AGC AGA AT TTTTT GAGGA CTAAA GACTT TCGGC TACAG AGGCT TT
SNP20545croAD1-A				GTC GCT CTC TCA AGC AGA AT TTTTT CGTTA ATATT TTGTT AATAT TTAAA TTGTA AA
SNP20545croAL6-A				GTC GCT CTC TCA AGC AGA AT TTTTT AGCTG ATTGC CCTTC ACAGT GAGAC GGGCA AC
SNP20545croAR1-A				GTC GCT CTC TCA AGC AGA AT TTTTT GTTAA ATAAG AATAA AGTGT GATAA ATAAG GC
SNP20545triA65-G				GTG CTG ACA CGG CCT GAT CC TTTTT TGCTA TTTTG CACCC AGCTA CAATT TTGTT TTGAA GCCTT AAA
SNP20545triB65-G				GTG CTG ACA CGG CCT GAT CC TTTT CCTGA CGAGA AACAC CAGAA CGAGT AGGCT GCTCA TTCAG TGA
SNP20545triC65-G				GTG CTG ACA CGG CCT GAT CC TTTTT ACGTG GACTC CAACG TCAAA GGGCG AATTT GGAAC AAGAG TCC
SNP22534-G	rs76222657	G	22.5kb	CT AAC CTT GTG TCG TAG ACA TTTTT GGCAG AGGCC ATGTG GAAA G
SNP22534-A	rs76222657	A	22.5kb	GA CGA CTG ACG CGG ACA TTC TTTTT GGCAG AGGCC ATGTG GAAA A
SNP22534triA65-G				TGT CTA CGA CAC AAG GTT AG TTTTT TGCTA TTTTG CACCC AGCTA CAATT TTGTT TTGAA GCCTT AAA
SNP2253				TGT CTA CGA CAC AAG GTT AG TTTTT

4triB65-G				CCTGA CGAGA AACAC CAGAA CGAGT AGGCT GCTCA TTCAG TGA
SNP2253 4triC65-G				TGT CTA CGA CAC AAG GTT AG TTTTT ACGTG GACTC CAACG TCAAA GGGCG AATTT GGAAC AAGAG TCC
SNP2253 4croAU6-A				GAA TGT CCG CGT CAG TCG TC TTTTT GAGGA CTAAA GACTT TCGGC TACAG AGGCT TT
SNP2253 4croAD1-A				GAA TGT CCG CGT CAG TCG TC TTTTT CGTTA ATATT TTGTT AATAT TTAAA TTGTA AA
SNP2253 4croAL6-A				GAA TGT CCG CGT CAG TCG TC TTTTT AGCTG ATTGC CCTTC ACAGT GAGAC GGGCA AC
SNP2253 4croAR1-A				GAA TGT CCG CGT CAG TCG TC TTTTT GTTAA ATAAG AATAA AGTGT GATAA ATAAG GC
SNP2253 4triA65-A				GAA TGT CCG CGT CAG TCG TC TTTTT TGCTA TTTTG CACCC AGCTA CAATT TTGTT TTGAA GCCTT AAA
SNP2253 4triB65-A				GAA TGT CCG CGT CAG TCG TC TTTTT CCTGA CGAGA AACAC CAGAA CGAGT AGGCT GCTCA TTCAG TGA
SNP2253 4triC65-A				GAA TGT CCG CGT CAG TCG TC TTTTT ACGTG GACTC CAACG TCAAA GGGCG AATTT GGAAC AAGAG TCC
SNP2470 8-C	rs12218 299	C	24.7kb	AT TCT GCT TGA GAG AGC GAC TTTTT TGGAA ATGAA GGCAA ATCA C
SNP2470 8-T	rs12218 299	T	24.7kb	GG ATC AGG CCG TGT CAG CAC TTTTT TGGAA ATGAA GGCAA ATCA T
SNP2470 8croAU6-C				GTC GCT CTC TCA AGC AGA AT TTTTT GAGGA CTAAA GACTT TCGGC TACAG AGGCT TT
SNP2470 8croAD1-C				GTC GCT CTC TCA AGC AGA AT TTTTT CGTTA ATATT TTGTT AATAT TTAAA TTGTA AA
SNP2470 8croAL6-C				GTC GCT CTC TCA AGC AGA AT TTTTT AGCTG ATTGC CCTTC ACAGT GAGAC GGGCA AC
SNP2470 8croAR1-C				GTC GCT CTC TCA AGC AGA AT TTTTT GTTAA ATAAG AATAA AGTGT GATAA ATAAG GC
SNP2470 8croAU6-T				GTG CTG ACA CGG CCT GAT CC TTTTT GAGGA CTAAA GACTT TCGGC TACAG AGGCT TT

SNP24708croAD1-T				GTG CTG ACA CGG CCT GAT CC TTTTT CGTTA ATATT TTGTT AATAT TTAAA TTGTA AA
SNP24708croAL6-T				GTG CTG ACA CGG CCT GAT CC TTTTT AGCTG ATTGC CCTTC ACAGT GAGAC GGGCA AC
SNP24708croAR1-T				GTG CTG ACA CGG CCT GAT CC TTTTT GTTAA ATAAG AATAA AGTGT GATAA ATAAG GC

Supplementary Table 7| Sequences of PCR primers, sequencing primers, M-strands and M3' staple strands in shape IDs for site-specific labeling of polymorphic sites on p53 gene samples (chr17: 7577233-7589583).

Strands		Allete	Location	Sequences
Pri12L	5' Phosphorylated			CCATCTACTCCCAACCAC
Pri12R				AATGATGGGTCGTTTGATA
SPri1L				CCATCTACTCCCAACCAC
SPri1R				TACCACCATCCACTACAACTA
SPri2L				GCAGCAAAGAAACAAACA
SPri2R				TCACCCATCTACAGTCCC
SPri3L				GTCGGGCTTCTGTCCTTC
Spri3R				GTGGCACAACCATAATAACTC
SNP77407-A	rs12951053	A	0.2kb	AT TCT GCT TGA GAG AGC GAC TTTTT TGGAT GGGTA GTAGT ATGG A
SNP77407-C	rs12951054	C	0.2kb	GG ATC AGG CCG TGT CAG CAC TTTTT TGGAT GGGTA GTAGT ATGG C
SNP77407croAU6-A				GTC GCT CTC TCA AGC AGA AT TTTTT GAGGA CTAAA GACTT TCGGC TACAG AGGCT TT
SNP77407croAD1-A				GTC GCT CTC TCA AGC AGA AT TTTTT CGTTA ATATT TTGTT AATAT TTAAA TTGTA AA
SNP77407croAL6-A				GTC GCT CTC TCA AGC AGA AT TTTTT AGCTG ATTGC CCTTC ACAGT GAGAC GGGCA AC
SNP77407croAR1-A				GTC GCT CTC TCA AGC AGA AT TTTTT GTTAA ATAAG AATAA AGTGT GATAA ATAAG GC
SNP77407croAU6-C				GTG CTG ACA CGG CCT GAT CC TTTTT GAGGA CTAAA GACTT

				TCGGC TACAG AGGCT TT
SNP77407croAD1-C				GTG CTG ACA CGG CCT GAT CC TTTTT CGTTA ATATT TTGTT AATAT TTAAA TTGTA AA
SNP77407croAL6-C				GTG CTG ACA CGG CCT GAT CC TTTTT AGCTG ATTGC CCTTC ACAGT GAGAC GGGCA AC
SNP77407croAR1-C				GTG CTG ACA CGG CCT GAT CC TTTTT GTTAA ATAAG AATAA AGTGT GATAA ATAAG GC
SNP79472-C	rs1042522	C	2.2kb	GA CGA CTG ACG CGG ACA TTC TTTTT TGCTG GTGCA GGGGC CACG G
SNP79472-G	rs1042523	G	2.2kb	CT AAC CTT GTG TCG TAG ACA TTTTT TGCTG GTGCA GGGGC CACG C
SNP79472triA65-C				GAA TGT CCG CGT CAG TCG TC TTTTT TGCTA TTTTG CACCC AGCTA CAATT TTGTT TTGAA GCCTT AAA
SNP79472triB65-C				GAA TGT CCG CGT CAG TCG TC TTTTT CCTGA CGAGA AACAC CAGAA CGAGT AGGCT GCTCA TTCAG TGA
SNP79472triC65-C				GAA TGT CCG CGT CAG TCG TC TTTTT ACGTG GACTC CAACG TCAAA GGGCG AATTT GGAAC AAGAG TCC
SNP79472triA65-G				TGT CTA CGA CAC AAG GTT AG TTTTT TGCTA TTTTG CACCC AGCTA CAATT TTGTT TTGAA GCCTT AAA
SNP79472triB65-G				TGT CTA CGA CAC AAG GTT AG TTTTT CCTGA CGAGA AACAC CAGAA CGAGT AGGCT GCTCA TTCAG TGA
SNP79472triC65-G				TGT CTA CGA CAC AAG GTT AG TTTTT ACGTG GACTC CAACG TCAAA GGGCG AATTT GGAAC AAGAG TCC
SNP88560-G	rs17882227		11.3kb	GA CTG CCT CTC ACC CAC CAT TTTTT ACGAA AACAT ATGAT CACA G
SNP88560-A	rs17882228		11.3kb	AC GTT ACC GGA CCT TAG CGA TTTTT ACGAA AACAT ATGAT CACA A

SNP88560tri A65-G				ATG GTG GGT GAG AGG CAG TC TTTTT TGCTA TTTTG CACCC AGCTA CAATT TTGTT TTGAA GCCTT AAA
SNP88560triB 65-G				ATG GTG GGT GAG AGG CAG TC TTTTT CCTGA CGAGA AACAC CAGAA CGAGT AGGCT GCTCA TTCAG TGA
SNP88560triC 65-G				ATG GTG GGT GAG AGG CAG TC TTTTT ACGTG GACTC CAACG TCAAA GGGCG AATTT GGAAC AAGAG TCC
SNP88560tri A65-A				TCG CTA AGG TCC GGT AAC GT TTTTT TGCTA TTTTG CACCC AGCTA CAATT TTGTT TTGAA GCCTT AAA
SNP88560triB 65-A				TCG CTA AGG TCC GGT AAC GT TTTTT CCTGA CGAGA AACAC CAGAA CGAGT AGGCT GCTCA TTCAG TGA
SNP88560triC 65-A				TCG CTA AGG TCC GGT AAC GT TTTTT ACGTG GACTC CAACG TCAAA GGGCG AATTT GGAAC AAGAG TCC
